# Carboxylic ligands and their influence on the structural properties of PbTe quantum dots

**DOI:** 10.1371/journal.pone.0328972

**Published:** 2025-07-31

**Authors:** Svetlana Lyssenko, Michal Amar, Alina Sermiagin, Refael Minnes

**Affiliations:** Department of Physics, Ariel University, Ariel, Israel; The Chinese University of Hong Kong, HONG KONG

## Abstract

We present a low-cost, straightforward, and tunable hot-injection method for synthesizing PbTe quantum dots (QDs). By incorporating short-chain carboxylic acids—hexanoic acid (HexA), heptanoic acid (HepA), and acetic acid (AcA)—alongside oleic acid (OA), we controlled QD morphology and size within the range of 13–17 nm. The resulting QDs exhibited a well-defined cuboctahedral shape and a core-shell structure, consisting of a crystalline core and an amorphous shell. Morphology and growth behavior were strongly influenced by precursor composition, ligand ratio, and steric hindrance. Compared to QDs synthesized with longer-chain acids (lauric (LA), decanoic (DA), and octanoic acids(OctA)), which produced multiple shapes, the use of shorter ligands led exclusively to uniform cuboctahedral nanocrystals. PbTe QDs are typically reported as cubic when their size exceeds 10 nm. In contrast, our method consistently produces cuboctahedral structures in this size range. QDs were characterized by high-resolution transmission electron microscopy (HRTEM), energy-dispersive X-ray spectroscopy (EDS), X-ray diffraction (XRD), and X-ray photoelectron spectroscopy (XPS). These findings open a route toward controlled shape engineering of PbTe QDs for future applications in quantum optics, infrared detectors, and thermoelectrics.

## 1. Introduction

Over the past decade, quantum dots (QDs) have emerged as a prominent area of interest in both industry and research. These nanoscale materials find extensive applications in various fields such as photovoltaics [1–4], telecommunication [5,6], sensors [7,8], biology [9], optics [1,8,10], and electronics [1,2,8]. QDs are widely used due to their distinctive chemical and physical properties; notably, the quantum confinement effect [11,12].

PbTe QDs have the largest exciton Bohr radii (a_0_ = 46 nm), a high dielectric constant, and a very small band gap. The larger exciton Bohr radii of PbTe enable the synthesis of larger QDs while preserving the quantum confinement effect.

A hot injection method is commonly used for QD synthesis. In brief, this method involves a reaction between metal and chalcogenide precursors in the presence of a capping ligand [13]. A wide variety of capping ligands are utilized for Pb-chalcogenide QDs, such as primary amines, thiocyanate ions, and carboxylic acids. The types of ligands involved include L-type ligands (Lewis bases that donate two electrons), X-type ligands (anions that donate one electron), and Z-type ligands (Lewis acids that accept two electrons) [14,15]. Capping ligands play a crucial role in QD formation. Organic ligands influence the size, shape, and composition of the resulting QDs [16].

A hot-injection method is thought to be a bottom-up approach [17]. A bottom-up approach is a seed-mediated growth method that includes two different steps [18]. The first step is the formulation of seeds, and the second step is seed growth [18]. Seed-mediated growth method depends on various parameters, such as reaction temperature [19], reactant concentration [20], seed population [21–23], seed structure [24], and surfactants [25,26].

Many QD characteristics and properties significantly depend on their size and morphology [18]. QD growth control is thought to be a kinetically controlled process [16,27]. Additionally, the nanocrystal (NC) shape is a thermodynamically controlled process [16,27]. Nanoparticles have a tendency to reduce their surface energy and surface stress, which is a widely recognized phenomenon [28]. Moreover, surface energy plays an important role in nanoparticle shape formation [28,29]. During nanoparticle growth, in order to minimize the surface energy, the facets favor growing on high-index planes [29]. Further, in a ligand-involved synthesis, the ligand regulates the flow of the seed mediation and its subsequent growth [20]. Usually, in fast reactions, the ligands connect to the seed surface, decreasing its energy and slowing its growth [20]. In the kinetic regime, high-energy facets exhibit a faster growth rate compared to low-energy facets, and they gradually diminish as the NC grows. This results in NCs that are ultimately terminated by slow-growing, low-energy facets [16]. Conventionally, the morphology of a face-centered cubic (fcc) NC is dictated by the ratio, R, between the growth rate along the 〈100〉 directions and that along the 〈111〉 directions [29,30]. For rock-salt crystalline structures, the 〈100〉 facet has a lower surface energy than the 〈111〉 facet [27]. Moreover, it is commonly assumed that spherical NCs, smaller than 10 nm, contain high-index crystallography planes and have higher surface energy [29,30]. It is a well-known fact that size and structure modification play a big role in the QDs’ characteristics and photophysics [10].

This work serves as a continuation of our previous studies, conducted with OctA, DA, and LA [31]. The current study focuses on optimizing the synthesis process of PbTe QDs using HexA, HepA, and AcA as capping ligands. The purpose of this study is to develop a novel, controllable hot-injection synthesis method specifically tailored for producing PbTe quantum dots (QDs) with tunable sizes and shapes. By utilizing a combination of oleic acid and short-chain carboxylic ligands, we aim to address and overcome the natural tendency of PbTe QDs to form cubic structures when their size exceeds 10 nm [13]. This allows for the successful synthesis of cuboctahedral QDs within the 13–17 nm size range. The proposed method offers significant advantages, particularly in simplifying the layer fabrication process, which is essential for various technological applications [32]. Ultimately, this research seeks to enhance the versatility and broaden the potential applications of PbTe QDs, especially in optoelectronic devices and other cutting-edge technologies.

## 2. Experimental

### 2.1 Materials

Tellurium (99.999% powder), lead (Ⅱ) acetate trihydrate (99%), squalene (99%) were purchased from Acros Organics; Heptanoic acid (HepA, 98%), hexanoic acid (HexA, 99%) were purchased from Thermo Scientific, acetic acid (AcA, 99%) oleic acid (OA, technical grade 90%), trioctylphosphine (TOP, technical grade 90%) were purchased from Alfa Aesar; Ethanol 99.99% was purchased from Romical; Hexane AR was purchased from Bio-Lab.

### 2.2 PbTe QDs synthesis

PbTe QDs were prepared using a hot-injection method [13]. Typically, in a glove box, a stock solution of 0.75M trioctylphosphine telluride (TOP-Te) was stirred overnight. The final solution should be transparent and yellow. The solution was removed from the glove box, and just before the beginning of the reaction, it was connected to a nitrogen Schlenk line. The synthesis of the PbTe NCs was performed in a 50 mL three-necked round-bottom flask. One neck of the flask was connected to a thermocouple, and the second neck was stopped with a rubber septum. The neck in the middle was connected to a condenser, which was connected to a Schlenk line. Pb(OAc)_2_·3H_2_O (0.1M in ligand and solvent solution) was mixed with OA and HexA/HepA ligands (in different ratios) in squalene to prepare a lead-acid precursor. Different ratios between long (OA) and short ligands (HexA/HepA), ranging from 6:0–4:2 (OA to HexA/HepA), were tested. The reaction solution was stirred and heated to 40°C under a vacuum until the bubbling stopped. The temperature was raised to 100°C. The heating was continued for 45 min to produce a transparent solution. Afterward, the solution was heated to 180°C under an N_2_ atmosphere and kept at this temperature for 10 min. The color of the solution turned slightly yellow. Pre-prepared TOP-Te solution (6 mL of 0.75M, corresponding to a 1: 3 Pb: Te molar ratio) was rapidly injected into the lead-acid hot solution, which was vigorously stirred. Due to instant nucleation, the color of the solution quickly turned black. After the injection, the temperature dropped to 155–170°C. Following the injection step, the QDs were allowed to grow for two minutes. Later on, the reaction was quenched using a water bath.

A typical synthesis of 16.8 nm PbTe NCs contains 0.57 g Pb(OAc)_2_·3H_2_O (1.5 mmol), 5.5 mL OA, 0.5 mL HepA, and 9 mL of squalene at the first step of lead-acid precursor preparation. Evacuation of water and acetic acid content coming from the lead salt at 100°C for 45 minutes is followed by heating the reaction mixture to 180°C. Rapid injection of 6 mL of 0.75 M TOP-Te solution (4.5 mmol) was performed after 10 minutes at 180°C. The reaction temperature dropped to 164–165°C. Two minutes after the injection, the reaction was quenched by replacing the heating mantle with a water bath. The reaction mixture was washed with 8 mL of hexane in a 50 mL centrifuge tube when the temperature reached 30°C. The tube was centrifuged for 4 minutes at 4400 rpm in order to precipitate PbTe NCs. After centrifugation, 20 mL of ethanol were added, and the NCs were centrifuged for an additional 4 min at 4400 rpm. The supernatant was removed, and the precipitated NCs were redispersed in hexane. For further purification, the hexane solution of the NCs solution was filtered through 0.22 µm and 0.1µ µm filters.

### 2.3 PbTe QDs synthesis with AcA

In the standard PbTe QDs synthesis, the acetic acid is usually removed from the reaction in the vacuum step. In order to synthesize QDs with AcA, a few adjustments had to be made. At the beginning of the reaction, the short ligand was not added to the reaction mixture, and a vacuum was applied to remove the water and acetate originating in Pb(OAc)_2_·3H_2_O. After 20 minutes, the vacuum was replaced with N_2_, and the AcA was added. The heating at 100°C was continued for 25 minutes. The following procedure steps were identical to those described in the general procedure mentioned earlier.

### 2.4 Instrumentation

HRTEM imaging was conducted with a JEOL JEM-2100F microscope operating at 200 keV, coupled with a GATAN 894 US1000 camera. The images were captured using a GATAN 806 HAADF STEM detector. EDS analysis was carried out using a JEOL JEM-2100F TEM operating at 200 kV, equipped with an Oxford Instruments X-Max 65T SDD detector. The probe size during analysis was set to 1 nm. For EDS data analysis, AZtec software (v. 3.3) was employed, and quantitative analysis was conducted using the standardless Cliff−Lorimer method at an accelerating voltage of 200 keV.

ImageJ software was utilized for calculations related to the size and size distribution of nanocrystals (NC), with image processing performed using the threshold function. XRD patterns were recorded using an X’Pert Pro diffractometer (PANalytical) equipped with a Cu Kα radiation source (λ = 1.5406 Å) to determine the crystal structure of the quantum dots (QDs). The X-ray generator was operated at 40 kV and 30 mA. Scans were conducted over a 2θ range of 15° to 85°, with a scan speed of 2.5°/min and a step size of 0.03°. The XPS analysis was performed using XPS ESCALAB 250 (Thermo Fisher Scientific). The analysis was performed with a spot size of 650 µm and an energy step size of 1.0 eV. The instrument was operated in constant analyzer energy (CAE) mode with a pass energy of 200.0 eV.

## 3. Results and discussion

The standard hot injection method for QD synthesis consists of only oleic acid, and the basic morphology in this synthesis ends with a cubic shape. This synthesis will act as our reference and control group with a 12.6 ± 1.1 nm size (S1 Fig). Our work started by comparing the quantum dots (QDs) sizes and shapes to the reference synthesis.

Initially, we started our research with longer ligands such as octanoic acid (OctA), decanoic acid (DA), and lauric acid (LA) mixtures with OA in the following ratios: 0.5: 5.5, 1: 5, 1.5: 5.5, 2: 4. Our previous work discovered that the ratio between the ligands and OA plays a significant role in the QD characteristics. We observed that a morphology change occurs when the short ligand concentration increases. Moreover, the particles grow bigger in the same case and lose part of their crystallinity. These conclusions led us to investigate shorter carboxylic acids.

This paper investigates the impact of very short capping ligands, namely hexanoic acid (HexA), heptanoic acid (HepA), and acetic acid (AcA) mixtures with oleic acid (OA), on the size and morphology of PbTe QDs. The mixtures were in the ratios discussed above—higher volumetric ratios led to the synthesis collapse, just like a synthesis without OA.

We conducted the first group of experiments with HexA. The PbTe-HexA_0.5_/OA_5.5_, PbTe-HexA_1_/OA_5_, PbTe-HexA_1.5_/OA_4.5_, PbTe-HexA_2_/OA_4_ QDs were successfully synthesized in a HexA: OA volumetric ratios of 0.5: 5.5, 1: 5, 1.5: 5.5, 2: 4 respectively.

**Fig 1:**
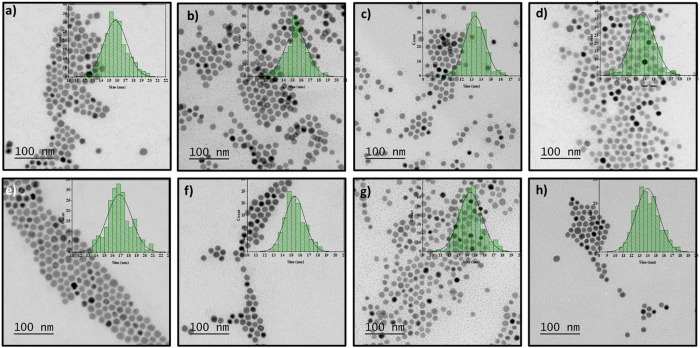
HRTEM images and their corresponding histograms. a) PbTe-HexA_0.5_/OA_5.5_, b) PbTe-HexA_1_/OA_5_, c) PbTe-HexA_1.5_/OA_4.5_, d) PbTe-HexA_2_/OA_4_, e) PbTe-HepA_0.5_/OA_5.5_, f) PbTe-HepA_1_/OA_5_, g) PbTe-HepA_1.5_/OA_4.5_, and h) PbTe-HepA_2_/OA_4_ QDs.

The first method for nanoparticle characterization was high-resolution scanning electron microscopy (HRTEM). HRTEM images were used to measure and calculate the QD’s size and identify the morphology of each synthesis. Fig 1a–1d represent the NCs in increasing order of HexA ligand volume and their corresponding histograms. The QD’s sizes were 15.8 ± 1.4, 16.2 ± 0.8, 13.3 ± 1.0, and 12.8 ± 1.0 nm for the discussed acid ratios, respectively. In this set of experiments, the standard deviation for all the nanoparticles is ≤ 8%. Moreover, a very narrow distribution can be observed for PbTe-HexA_1_/OA_5_ QDs with only a 5% standard deviation. This synthesis is the most uniform, and due to the high NCs’ uniformity, they may easily self-assemble into a superlattice. This effect is visible in Fig 1b and 1c for PbTe-HexA_1_/OA_5_, PbTe-HexA_1.5_/OA_4.5_ QDs. The small nanoparticles in this synthesis didn’t contribute to the discussed population of QDs. The morphology we detected was hexagonal-cuboctahedral, in contrast with the cubical shape in the reference synthesis. As one can observe, the size decreases with the increase in hexanoic acid. Energy-dispersive X-ray spectroscopy (EDS) analysis did not detect any change in the atomic ratio. In all the experiments mentioned, the EDS ratio between Pb and Te was ~ 1.20: 1 (S1 Table). S2 Fig shows the EDS spectra.

Fig 1e–1h show the synthesized QDs in different ratios of HepA to OA (PbTe-HepA_0.5_/OA_5.5_, PbTe-HepA_1_/OA_5_, PbTe-HepA_1.5_/OA_4.5_, PbTe-HepA_2_/OA_4_ QDs) with the corresponding sizes of 16.8 ± 1.5, 15.3 ± 1.1, 14.4 ± 1.3, and 13.9 ± 1.4 nm, respectively. The standard deviation of ≤ 10% shows a narrow size distribution, pointing out the particles’ uniformity.

Consistent results were observed according to the QDs’ morphology; similar shapes were distinguished as in the previous experiments with HexA.The atomic ratios of Pb: Te, calculated from EDS analysis, are 1.10: 1, 0.95: 1, 0.88: 1, and 0.8: 1. A slight decrease in the Pb element appeared. The EDS ratio between Pb and Te elements for small nanoparticles is 2.9: 1. Small nanoparticles mainly consist of the Pb element, which may explain the decrease in the Pb percentage in the big NCs. All the calculated EDS ratios are listed in S1 Table, and the EDS spectra are presented in S3 and S4 Figs.

The last set of experiments involved different ratios of AcA to OA. However, only one of the experiments was successful with the acid ratios of 1:5, PbTe-AcA_1_/OA_5_. The size histogram and shape of the QDs can be found in S5 Fig. In this synthesis, at least two populations of nanoparticles were observed with sizes of ~9 nm and ~16 nm. It is also important to mention that this synthesis is unstable over time. The EDS analysis was performed in the NC center and at the NC edge (S6 Fig). The Pb: Te atomic ratios were 0.87: 1 and 3.32: 1 for the center and the edge, respectively (S1 Table). The difference in the composition of the elements indicates a core-shell formation. The same phenomena were observed in our previous work. Based on the results presented in this part, we can assume that those PbTe-AcA/OA QDs are unstable due to the high reactivity of the AcA ligand and low QDs surface passivation ability.

**Fig 2:**
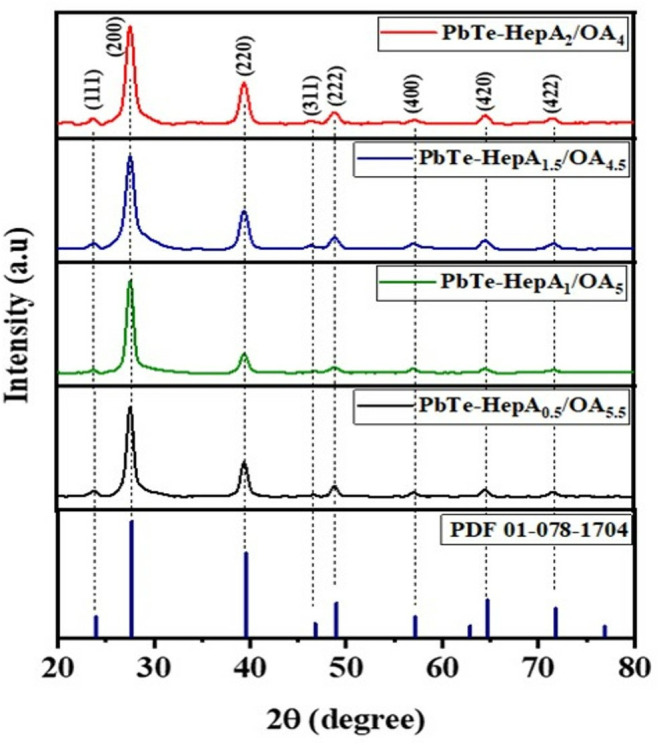
XRD spectra of PbTe QDs. XRD spectra of PbTe-HepA/OA QDs and PbTe pattern from the database -PDF 01-078-1904 (blue).

X-ray diffraction (XRD) was used to test the samples. All the PbTe QDs mentioned above showed a cubic crystalline structure with an Fm-3m space group and a lattice constant of a = 6.438 Å (PDF 01-078-1704). The synthesized QDs with AcA weren’t stable enough for XRD measurements. The XRD peaks at 2Ɵ degree angles were obtained at 23.75, 27.58, 39.44, 46.38, 48.85, 57.13, 64.51, and 71.8. These 2Ɵ angles correlate with [111], [200], [220], [222], [400], [420], and [422] crystallite planes (Figs 2 and S7). All the planes were also detected in the reference synthesis (S1 Fig). From these measurements, we may conclude that the ligands have no apparent effect on the crystalline structure of the QDs. However, it has been previously reported that in some cases, the ligands play a significant role in the crystalline QDs structure [33,34].

**Fig 3:**
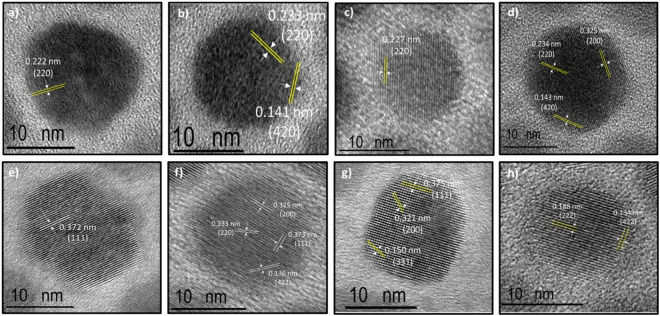
High magnification HRTEM images and their corresponding d-spacing and hkl indexes. a) PbTe-HexA_0.5_/OA_5.5_, b) PbTe-HexA_1_/OA_5_, c) PbTe-HexA_1.5_/OA_4.5_, d) PbTe-HexA_2_/OA_4_, e) PbTe-HepA_0.5_/OA_5.5_, f) PbTe-HepA_1_/OA_5_, g) PbTe-HepA_1.5_/OA_4.5_, and h) PbTe-HepA_2_/OA_4_ QDs.

Following the XRD analysis, d-spacing calculations were carried out on HRTEM images using the FFT function of ImageJ software. The measured d-spacings were 0.366, 0.320, 0.229, 0.197, 0.182, 0.160, 0.148, 0.142, and 0.129 nm; the corresponding hkl indexes are in line with XRD analysis. The d-spacings of each synthesis are shown in Fig 3a–3h and S2–S10 Tables. The measured d-spacings, facets, and 2Ɵ angles of all the PbTe QDs are repetitive, constant, and known from the literature [29,35–37].

The crystallite size was obtained from the [200] peak of the XRD pattern using the Scherrer equation (Table 1) [38]. The XRD NC sizes range between 8 and 10 nm and are much smaller than the HRTEM sizes. However, based on our previous results, we expected to achieve a core-shell structure with a small crystalline core size. The difference between XRD and HRTEM sizes comes from the shell thickness. The calculated shell width is approximately 3 nm.

**Table 1:** The PbTe NCs sizes measured by HRTEM and XRD.

Short Acid	A/OA ratio	HRTEM size (nm)	XRD NC size (nm)
HexA	0.5/5.5	15.8 ± 1.4	8.94
	1/5	16.2 ± 0.8	9.40
	1.5/4.5	13.3 ± 1.0	8.13
	2/4	12.8 ± 1.0	8.42
HepA	0.5/5.5	16.8 ± 1.5	9.44
	1/5	15.3 ± 1.1	9.82
	1.5/4.5	14.4 ± 1.3	9.78
	2/4	13.9 ± 1.4	8.08
AcA	1/5	8.82/16.4˟	****

˟ represents two different NC population sizes.

**Fig 4:**
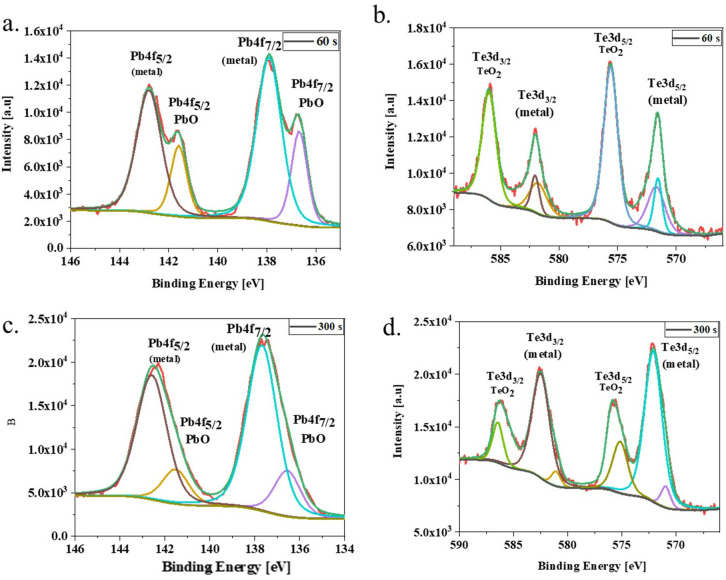
XPS high-resolution survey spectrum for PbTe-HexA/OA QDs. a) Pb4f peaks at surface level- 60s, b) Te3d at surface level- 60s, c) Pb4f peaks at core level- 300s, d) Te3d at core level- 300s.

X-ray photoelectron spectroscopy (XPS) was utilized to investigate the elemental composition and chemical oxidation states of the elements within the PbTe QDs. A complete XPS survey is provided in S8 Fig. The survey spectrum shows all the relevant peaks for Pb-4f, Te-3d, and O-1s, which is in line with the literature [39]. Pb-4f and Te-3d showed doublets due to spin-orbit coupling [40]. A surface and core XPS analysis was conducted in all PbTe QD samples to distinguish the core-shell structures. The high-resolution spectra for HexA-QDs and HepA-QDs are presented in Figs 4 and S9, respectively. Additionally, no XPS analysis has been done due to the high instability of AcA-QDs.

In HepA-QDs measurements, we detected Pb-4f peaks in the binding energy of 136-140eV for Pb-4f7/2 and 142-146eV for Pb-4f5/2. Four different peaks were detected; two represented Pb^0^-metal, and the other two were of Pb-oxide. The surface (60s) and core (300s) analysis showed a difference in the intensities of these peaks. The surface analysis showed low amounts of Pb-oxides. However, the core analysis detected a significant increase in Pb-oxides. Further high-resolution XPS analysis shows Te-3d3/2 and Te-3d5/2 peaks centered at 580-586eV and 569-576eV binding energies, respectively. The tellurium analysis showed the Te-oxide peaks were higher than the Te^0^-metal in both surface and core levels. An increase in both Te-peaks was detected. However, the ratio of the peaks remained the same.

Following the HepA-QDs analysis, HexA-QDs were tested, and the high-resolution XPS analysis showed Pb-4f5/2 and pb-4f7/2 peaks centered at 140-145eV and 135-139eV, respectively. In both surface and core levels, the Pb^0^-metal peaks were higher, and the Pb-oxides were lower. The tellurium measurements for Te-3d3/2 and Te-3d5/2 peaks were centered at 580-589eV and 570-576eV, respectively. In this case, it is easy to determine higher Te-oxide peaks at the surface level, which decrease with in-depth analysis. The core level showed much higher Te^0^-metal peaks. A suboxide peak of tellurium can also be detected in this synthesis at both surface and core levels.

It is essential to mention that all the Pb-4f5/2 and pb-4f7/2 metal peaks are higher than the oxides in all surface measurements for all the measured ligands in this paper, as well as the previous one [32]. However, when we compare the surface/core-level measurements of all the different synthesizes, a clear conclusion can be made- if a high number of small nanoparticles can be detected in the QD synthesis, then measurements show more oxide species all over the measured analysis.

XPS excels at revealing a QD’s overall composition, including surface ligands, shell coverage, and surface oxidation. However, its usefulness in deciphering finer details is limited [41]. XPS analyzes a depth comparable to the QD itself, blurring the line between surface and core information. Additionally, the minimum XPS spot size is much larger than individual QDs, hindering its ability to probe specific features like surface bonding or core-shell interfaces within these tiny structures.

In our previous work, we synthesized PbTe QDs with mixtures of OA/OctA (C8), OA/DA (C10), and OA/LA (C12). Our results are in line with the literature, and they show that whenever we synthesize the QDs with OA alone, the morphology is cubical for NCs bigger than 10 nm [13]. However, the addition of a short ligand stimulates a morphology change and leads to cuboctahedral shapes even in QDs of 10 nm and higher. Furthermore, we found out that with the increase in the short acid volume, the QDs’ size increases. Also, our former results showed that with the addition of a shorter ligand (OctA), a morphology change occurs at lower concentrations compared to DA and LA.

Our ligands in this study, namely AcA – C2, HepA – C5, and HexA – C6, are considerably shorter and exhibit higher reactivity compared to the ligands used in previous studies. AcA is the shortest ligand in this case, whereas OctA was the shortest ligand in our previous work. Our initial set of experiments conducted with HexA showed that with a high HexA/OA volumetric ratio, the NCs’ size decreased from approximately 16–13 nm. We did not observe a morphology change during our synthesis, and from the very beginning, the morphologies of the QDs were cuboctahedral and monodispersed. Moreover, the QD yield becomes lower with increasing concentration. Consequently, in high ligand ratios such as 3: 3, the NCs fold onto themselves during the synthesis quenching step. We noticed the same trend for PbTe-HepA/OA QDs. The QD’s size decreased from approximately 17–14 nm.

The synthesis steps with AcA are a bit different, and for this reason, the results are unlike all the other short ligands. A standard hot injection method with Pb-acetate salt usually involves a vacuum step. During this step, acetate molecules are disposed of, and the Pb-ligand complex evolves. In the AcA case, the acid was added in a controlled manner. The AcA synthesis is unstable, and in many cases, the NCs agglomerate, precipitate, or don’t evolve at all. Several reasons can contribute to the lack of stability, including the QD’s poor surface passivation, high acid reactivity, and low-temperature stability.

In each synthesis, a small nanoparticle population was observed in addition to the main population of the QDs. These nanoparticles are mostly composed of 75% Pb and only 25% Te. We assume that the nanoparticles evolved from the excess of the Pb-precursor that did not take part in the formation of the larger population. Therefore, we may also assume that we have a Te-precursor deficiency. Moreover, the presence of the small nanoparticles does not affect the QDs’ formation, growth, or monodispersity in the beginning. The addition of the second acid may lead to the formation of a higher number of nuclei, which later leads to the creation of a large population of small nanoparticles. The nuclei number is a direct result of the high short-acid reactivity. In a later step, at higher acid concentrations, we observed that the small nanoparticle population started to grow, and the main population yield was reduced.

**Fig 5:**
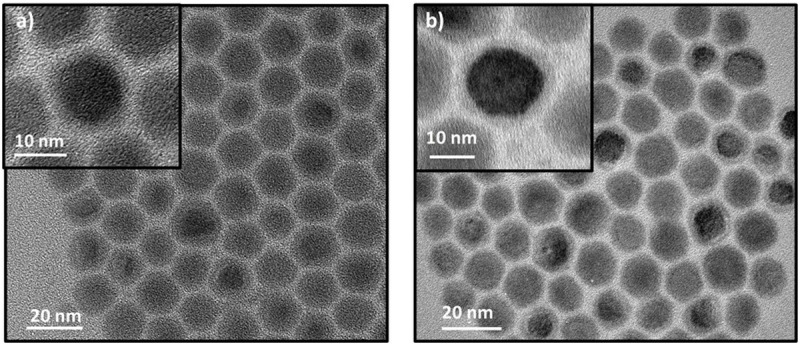
HRTEM images. a) PbTe-Hep_1_A/OA_5_ and b) PbTe-Hex_1_A/OA_5_ QDs, with insets highlighting a single particle exhibiting a core-shell structure.

All the presented results show that the addition of a second acid changes the supersaturation and the nuclei number. We also observed that all the synthesized QDs, in this case, are much bigger than the NCs in the reference synthesis (PbTe-OA QDs). However, in these short ligands, we noticed a new, interesting phenomenon. In PbTe-HexA_0.5_/OA_5.5_ and HexA_1_/OA_5,_ 16 nm NCs were discovered in HRTEM, and the more HexA we added, the smaller the particles became. We observed the same phenomenon in HepA. Nevertheless, when the same QDs were analyzed by XRD, it was found that the NCs’ size was smaller and constant around 9 nm (Table 1). In our opinion, the size difference is related to the core-shell structure, when the core is crystalline, and the shell is amorphous (Fig 5). Fig 5 demonstrates some of the core-shell structures of PbTe-Hep_1_A/OA_5_ and PbTe-Hex_1_A/OA_5_ QDs.

Literature indicates that an amorphous shell could consist of oxides [42]; however, we strongly believe that the amorphous shell observed in our samples is not primarily due to oxidation. Furthermore, studies suggest that an amorphous shell formed through oxidation would typically recrystallize under the electron beam during HRTEM analysis [43], which we do not observe in our case. Our findings demonstrate that the shell remains stable even after imaging the same particles three times at increasing magnifications (S10 Fig).

Following the continuous research into different short ligands, we can now distinguish between two ligand groups. The initial group, group A, consists of OctA, DA, and LA. The second group, group B, is HexA and HepA. Those groups do not include AcA. Group A showed a morphology change, and the NCs grew with the increase in short ligand concentration. In comparison, in group B, a morphology change wasn’t detected, and the QDs got smaller with the short acid concentration increase. Moreover, it was observed that morphology change in OctA occurred at lower acid ratios (OctA_1_: OA_5_) compared to DA, where the morphology transformed at higher rations (1.5: 4.5). However, the morphology change with group B, which includes the shortest ligands, starts even at lower ratios than 0.5: 5.5 and cannot be detected. The shorter and more reactive the ligand, the more influence it has on the QD shape transformation. The reference synthesis with OA shows 12.6 nm cubical QDs. It is a well-known fact that the PbTe QDs are oleic acid-dependent and have a high structural attachment to the utilized acid [11]. In the synthesis with OA, the ligand often exhibits {111} and {100} facets that are approximately equal in size, leading to a cubical shape [44,45].

According to the literature, in the field of nanoparticle science, the formation of the nanoparticle is determined by two primary regimes: kinetic and thermodynamic. The thermodynamic growth regime requires a sufficient temperature supply. In this regime, monomers flow slowly, and stable cube crystallite structures are typically preferred. However, in the kinetic growth regime, a high flow of monomers occurs, and high-index facet structures are favored. Dong Y. et al. showed that the more oleic acid added to the reaction, the fewer nuclei formed [46]. When the OA concentration is reduced, the QDs grow bigger [46]. In other words, the increasing concentration of OA is correlated with kinetic control. The addition of short ligands makes the kinetics even more pronounced.

In our case, we lower the concentration of OA, which is supposed to lead to big nuclei, resulting in big QDs. However, in both of our research cases, groups A and B, we observe two opposite trends. In the group A experiments, the NCs grow bigger at higher acid ratios. However, in group B experiments, big NC evolved at very low acid ratios (0.5:5.5), and the NC became smaller with the increase of the short acid volume. The big NC from the beginning leads us to believe that a modification in seed formation occurs. Following these results, we assume that when the ligand is long, the nuclei grow more slowly than in short ligand cases. Meaning, the reaction is controlled by its kinetics. In group B, the reaction is under kinetic and thermodynamic control. In both cases, the kinetic is more pronounced. The final NC morphology is dependent on the balance between kinetics and thermodynamics.

The differences between the chosen ligands in group A are not significant enough to lead to a change from kinetic to thermodynamic control. The acids in group B are significantly shorter compared to the ligands in group A. Group B has short ligands that are fundamentally shorter, more reactive, and have a high diffusion coefficient. The acid flux is higher and more dependent on temperature than the acids from group A. On the one hand, seed formation and growth depend on the rate of Pb-ligand absorption. Short ligands direct the formation in a fast manner, resulting in a higher number of small seeds. However, longer ligands slow down seed formation and create bigger, more stable seeds. Kinetics has a greater impact on the process of seed formation.

The EDS atomic ratio of Pb: Te in all NCs’ cores is mostly 1: 1. Nevertheless, the shell atomic composition is 75% Pb and only 25% Te. Based on these results, we assume that the growth of the nanoparticles after the seeds’ formation step is related to the monomers (Pb-carboxylic acid and TOP-Te) supplied to the seeds. The size of the NCs increases until the monomer concentration is decreased, the monomer flux is reduced, and the steric hindrance becomes more significant. TOP is much more sterically hindered compared to OA and any other shorter ligand; hence, TOP-Te supply to the NC seed is much slower.

From the literature, it is well known that Pb-oleate, a Z-type ligand, accepts two electrons [47,48]. We estimate that as the monomer concentration decreases, the Pb-complex begins to act as a Z-type ligand. This shift in ligand behaviour affects QD morphology, leading to a transformation from the expected cubic shape to a cuboctahedral form. According to the Wulff construction, the cubic shape generally represents the final growth stage for nanocrystals [49]. We assume that the QDs halt growth at this intermediate cuboctahedral stage, with further growth occurring through shell expansion that maintains the crystalline core’s shape. Jeong D. et al. found that the Z-type ligand promotes NC growth [50]. However, such core/shell structures are not extensively documented in the literature, and we cannot fully support our assumptions without further investigation. Further research is needed to fully understand the formation of the amorphous shell in such core/shell structures. This additional investigation will be essential to elucidate the mechanisms driving shell growth and the factors influencing its amorphous nature.

Following this line of thought, the QDs from group A increase in size with the addition of the short acid ligands. In comparison, the QDs from group B start as big particles and decrease in size with the addition of the short ligand acids. These two opposite trends can be easily explained by the short ligand reactivity, mobility, and the small nanoparticle population growth. As previously stated, a higher reactivity of the ligand leads to an increase in the number of nuclei. Group A shows that the monomers attach to the core and increase the thickness of the shell, which increases the QD size. Therefore, high short-ligand concentrations lead to a thicker shell, hence bigger QDs. However, in group B, ligands are much shorter and lead to the formation of a higher number of nuclei compared to group A. The increase in the short ligand volume leads to an increase in the small nanoparticle population. The small nanoparticle population steals the monomers from the main QDs’ population and consequently grows, leading to a decrease in the size of the main QDs population.

## 4. Conclusions

This study presents a straightforward, low-cost, and tunable hot-injection method for synthesizing PbTe QDs with controlled size and morphology. By partially replacing OA with shorter carboxylic acids—HexA, HepA, and AcA—we achieved uniform cuboctahedral QDs with sizes ranging from 13 to 17 nm. We propose a growth mechanism explaining the formation of a crystalline core and an amorphous shell, influenced by ligand reactivity and precursor composition.

Our findings demonstrate that QD shape evolution follows similar stages for both ligand groups A and B; however, the resulting sizes differ due to variations in nucleation dynamics. A higher number of nuclei leads to smaller QDs, emphasizing the role of acid reactivity and steric effects in growth control.

This synthesis approach offers reproducible control over structural features critical for applications requiring uniform morphology, such as layer-by-layer assemblies. The cuboctahedral shape, in particular, enhances packing density and surface interaction, contributing to the stability and performance of nanostructured films. These results contribute valuable insights into the structure–property relationship in QD systems and provide a foundation for further development of scalable, shape-directed nanomaterial fabrication methods.

## Supporting information

S1 Fig HRTEM images and XRD spectra. a) HRTEM images of cubic PbTe-OA QDs of 12.6 ± 1.1 nm size and its corresponding histogram and b) XRD pattern of cubic PbTe-OA QDs 12.6 nm (red) and PbTe pattern from the database -JCPDS 01-078-1904 (blue).
(JPG)

S2 Fig EDS spectrum. a) PbTe- HexA_0.5_/OA_5.5_ QDs, b) PbTe- HexA_1_/OA_5_ QDs, c) PbTe- HexA_1.5_/OA_4.5_ QDs, and d) PbTe- HexA_2_/OA_4_ QDs.
(JPG)

S3 Fig EDS spectrum. a) PbTe-HepA_0.5_/OA_5.5_ QDs, b) PbTe-HepA_1_/OA_5_ QDs, c) PbTe-HepA_1.5_/OA_4.5_ QDs, and d) PbTe-HepA_2_/OA_4_ QDs.
(JPG)

S4 Fig EDS analysis. a) EDS spectrum b) image of the measured EDS area of small nanoparticles evolved in PbTe-HepA_1.5_/OA_4.5_ QDs synthesis.
(JPG)

S5 Fig HRTEM image and its corresponding histogram of PbTe-AcA_1_/OA_5_ QDs. (JPG)

S6 Fig EDS analysis. a) EDS spectrum PbTe-AcA_1_/OA_5_ QDs core, b) EDS spectrum of PbTe-AcA_1_/OA_5_ QDs and, and c) image of the corresponding measured EDS areas.
(JPG)

S7 Fig XRD spectra of PbTe-HexA/OA QDs and PbTe pattern from the database – PDF 01-078-1904 (blue). (JPG)

S8 Fig XPS survey spectrum for PbTe QDs. (JPG)

S9 Fig XPS high resolution survey spectrum for PbTe-HepA/OA QDs. a) Pb4f peaks at surface level- 60s, b) Te3d at surface level- 60s, and c) Pb4f peaks at core level- 300s, d) Te3d at core level- 300s.
(JPG)

S10 Fig HRTEM images of core-shell PbTe-Hep_1_A/OA_5_ QDs taken at different magnifications. (JPG)

S1 Table Atomic % of Pb and Te elements and Pb: Te ratio in PbTe QDs of all the experiments. (PDF)

S2 Table d – spacing calculations. d – spacing of PbTe-HepA_0.5_/OA_5.5_ calculated from HRTEM images and its corresponding hkl index.
(PDF)

S3 Table d – spacing calculations. d – spacing of PbTe-HepA_1_/OA_5_ calculated from HRTEM images and its corresponding hkl index.
(PDF)

S4 Table d – spacing calculations. d – spacing of PbTe-HepA_1.5_/OA_4.5_ calculated from HRTEM images and its corresponding hkl index.
(PDF)

S5 Table d – spacing calculations. d – spacing of PbTe-HepA_2_/OA_4_ calculated from HRTEM images and its corresponding hkl index.
(PDF)

S6 Table d – spacing calculations. d – spacing of PbTe-HexA_0.5_/OA_5.5_ calculated from HRTEM images and its corresponding hkl index.
(PDF)

S7 Table d – spacing calculations. d – spacing of PbTe-HexA_1_/OA_5_ calculated from HRTEM images and its corresponding hkl index.
(PDF)

S8 Table d – spacing calculations. d – spacing of PbTe-HexA_1.5_/OA_4.5_ calculated from HRTEM images and its corresponding hkl index.
(PDF)

S9 Table d – spacing calculations. d – spacing of PbTe-HexA_2_/OA_4_ calculated from HRTEM images and its corresponding hkl index.
(PDF)

S10 Table d – spacing calculations. d – spacing of PbTe-AcA_1_/OA_5_ calculated from HRTEM images and its corresponding hkl index.
(PDF)
