# Supplementary figures and images for: Carboxylic ligands and their influence on the structural properties of PbTe quantum dots
Source: PLoS One. 2025 Jul 31;20(7):e0328972. doi: 10.1371/journal.pone.0328972 (PMC12312907; doi:10.1371/journal.pone.0328972)

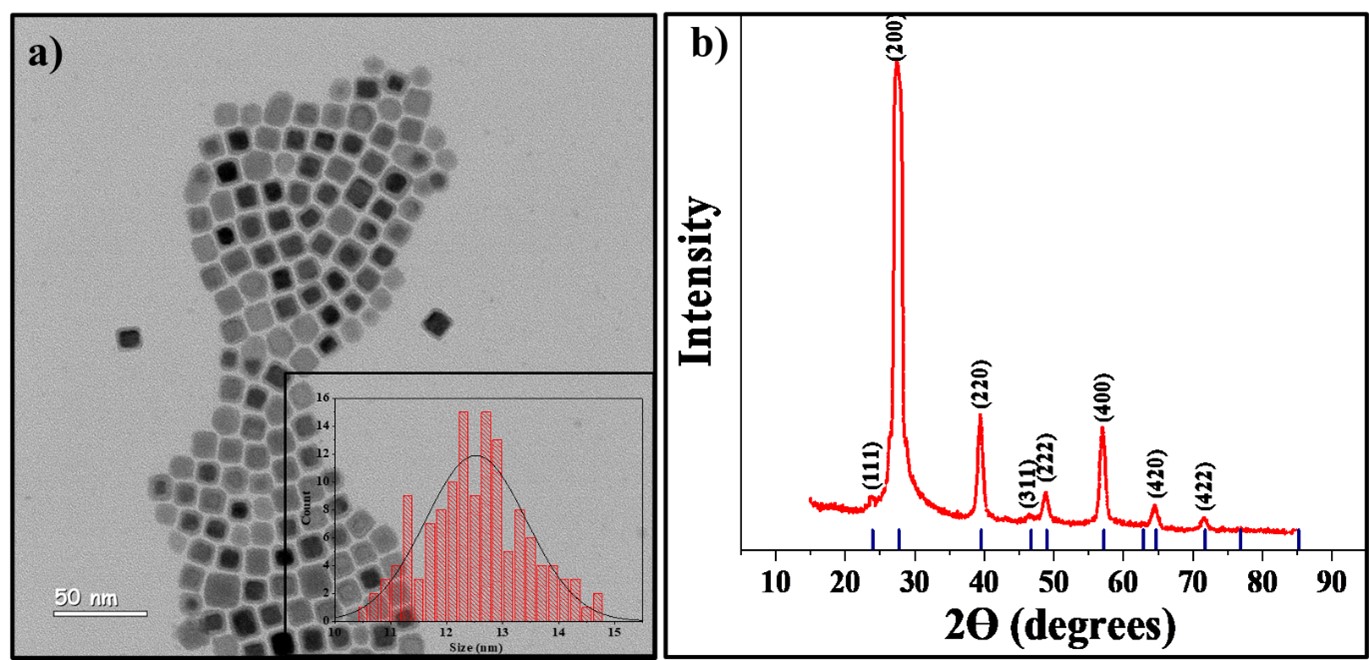

Supplement: S1 Fig — a) HRTEM images of cubic PbTe-OA QDs of 12.6 ± 1.1 nm size and its corresponding histogram and b) XRD pattern of cubic PbTe-OA QDs 12.6 nm (red) and PbTe pattern from the database -JCPDS 01-078-1904 (blue). (JPG) [file pone.0328972.s001.jpg]

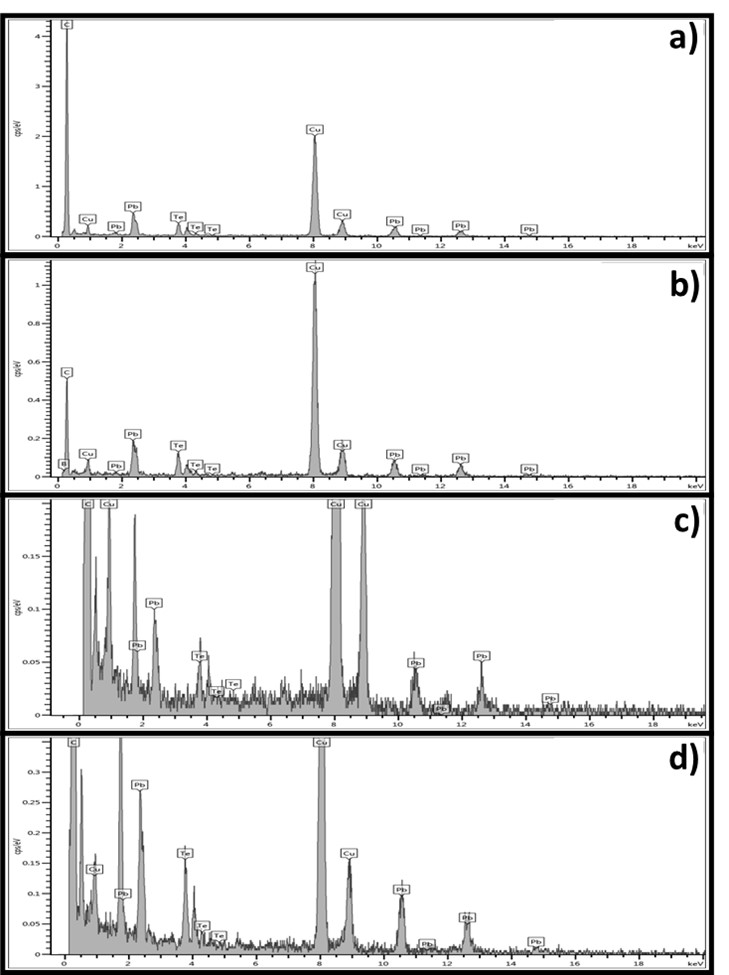

Supplement: S2 Fig — a) PbTe- HexA0.5/OA5.5 QDs, b) PbTe- HexA1/OA5 QDs, c) PbTe- HexA1.5/OA4.5 QDs, and d) PbTe- HexA2/OA4 QDs. (JPG) [file pone.0328972.s002.jpg]

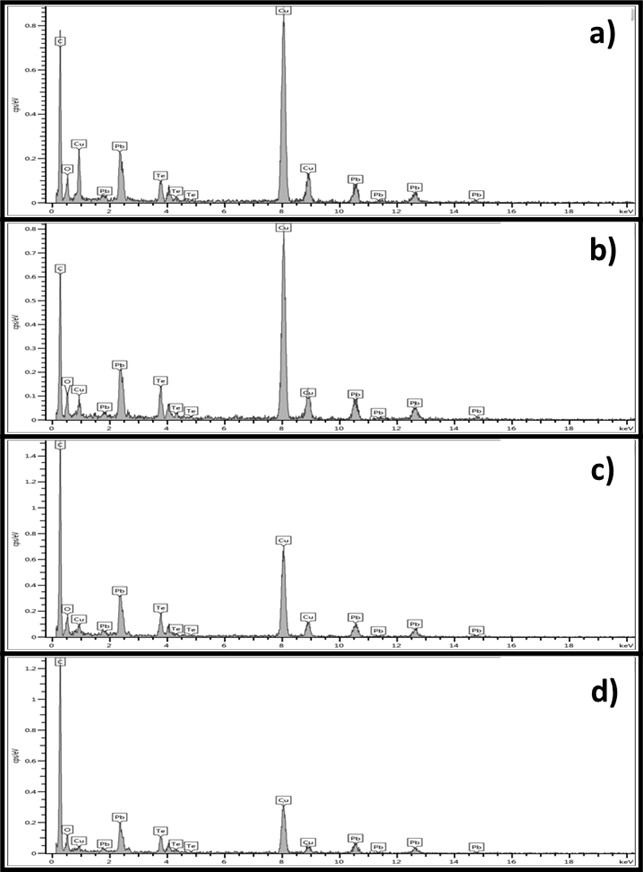

Supplement: S3 Fig — a) PbTe-HepA0.5/OA5.5 QDs, b) PbTe-HepA1/OA5 QDs, c) PbTe-HepA1.5/OA4.5 QDs, and d) PbTe-HepA2/OA4 QDs. (JPG) [file pone.0328972.s003.jpg]

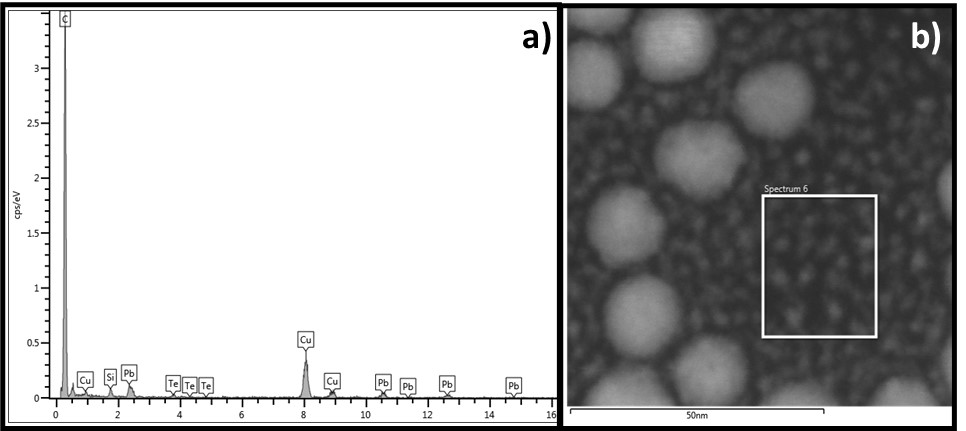

Supplement: S4 Fig — a) EDS spectrum b) image of the measured EDS area of small nanoparticles evolved in PbTe-HepA1.5/OA4.5 QDs synthesis. (JPG) [file pone.0328972.s004.jpg]

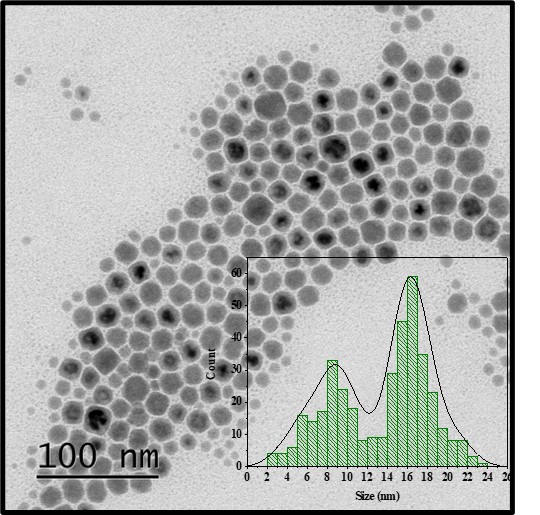

Supplement: S5 Fig — (JPG) [file pone.0328972.s005.jpg]

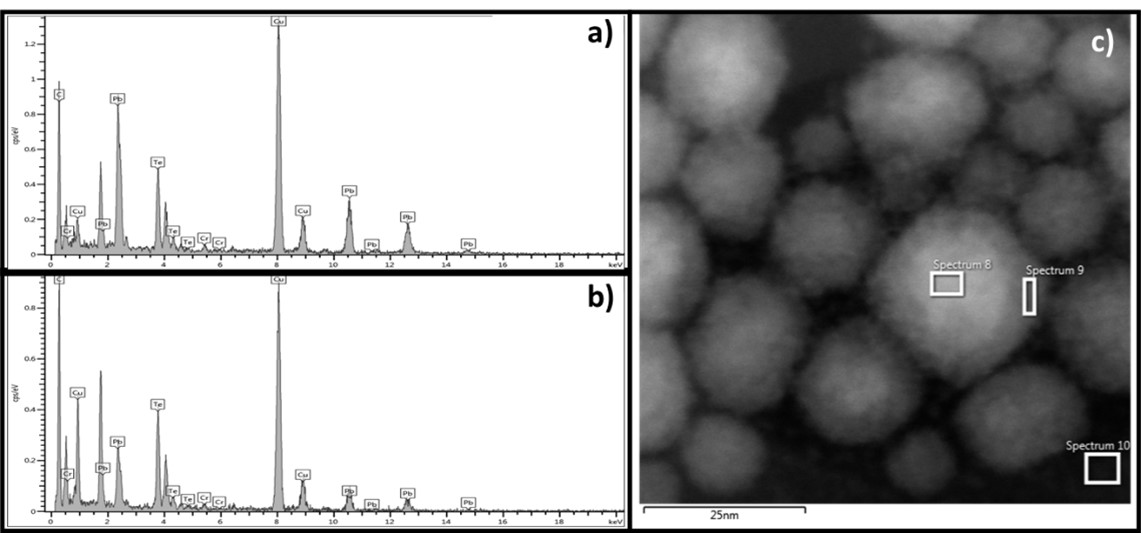

Supplement: S6 Fig — a) EDS spectrum PbTe-AcA1/OA5 QDs core, b) EDS spectrum of PbTe-AcA1/OA5 QDs and, and c) image of the corresponding measured EDS areas. (JPG) [file pone.0328972.s006.jpg]

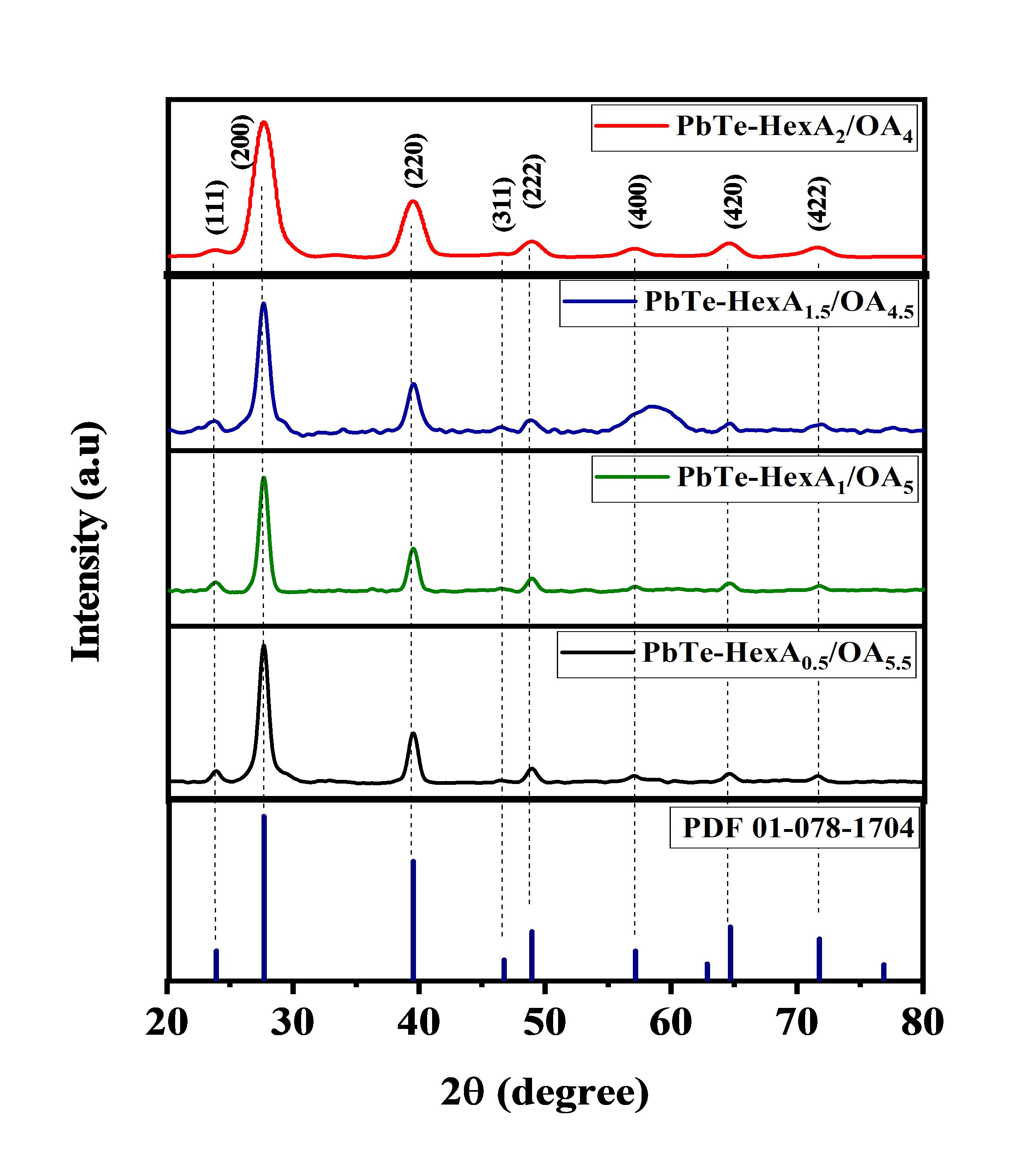

Supplement: S7 Fig — (JPG) [file pone.0328972.s007.jpg]

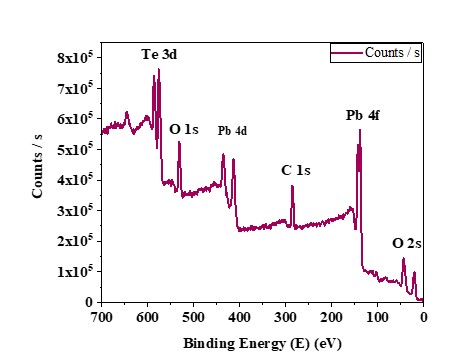

Supplement: S8 Fig — (JPG) [file pone.0328972.s008.jpg]

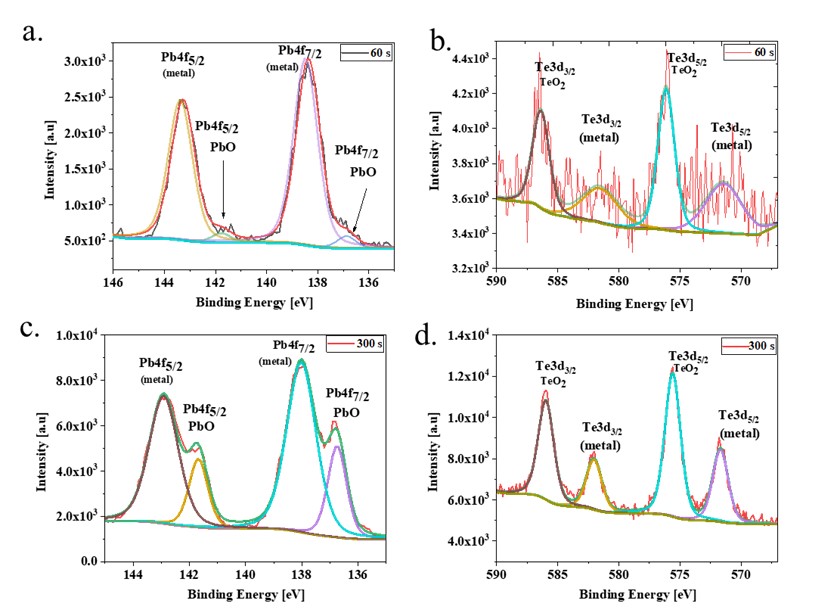

Supplement: S9 Fig — a) Pb4f peaks at surface level- 60s, b) Te3d at surface level- 60s, and c) Pb4f peaks at core level- 300s, d) Te3d at core level- 300s. (JPG) [file pone.0328972.s009.jpg]

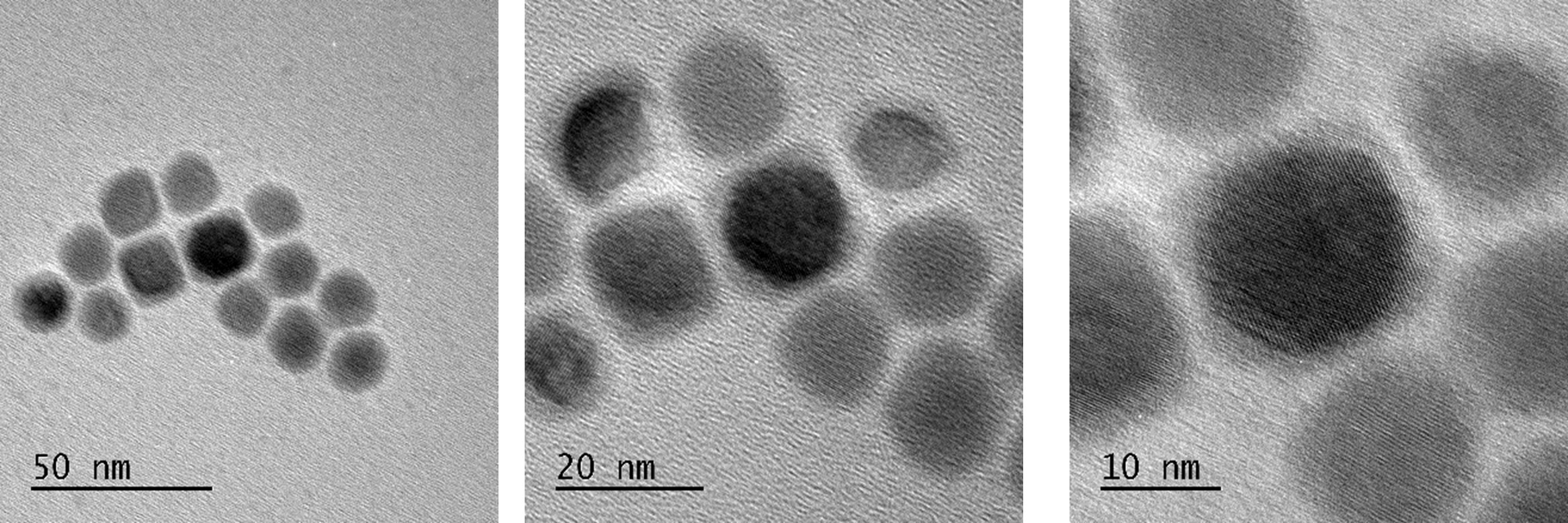

Supplement: S10 Fig — (JPG) [file pone.0328972.s010.jpg]
