# Supplementary material for: Carboxylic ligands and their influence on the structural properties of PbTe quantum dots
Source: PLoS One. 2025 Jul 31;20(7):e0328972. doi: 10.1371/journal.pone.0328972 (PMC12312907; doi:10.1371/journal.pone.0328972)
Supplement: S1 Table — (PDF) [file pone.0328972.s011.pdf]

Table S1. Atomic % of Pb and Te elements and Pb: Te ratio in PbTe QDs of all the experiments.

Acid Type	Acids Ratio	Atomic %		Atomic % Ratio
		Pb	Te	Pb:Te
HexA	0.5\5.5	51.34	48.66	1.05
	1\5	54.54	45.46	1.19
	1.5\ 4.5	55.3	44.7	1.23
	2\4	54.14	45.86	1.18
HepA	0.5\5.5	52.18	47.82	1.1
	1\5	48.39	51.05	0.95
	1.5\ 4.5	46.94	53.06	0.88
	2\4	44.37	55.63	0.8
	1.5\ 4.5	74.45	25.55	2.9
AcA	1\5 core	46.48	53.52	0.87
	1\5 shell	76.87	23.13	3.32
