# Supplementary material for: Carboxylic ligands and their influence on the structural properties of PbTe quantum dots
Source: PLoS One. 2025 Jul 31;20(7):e0328972. doi: 10.1371/journal.pone.0328972 (PMC12312907; doi:10.1371/journal.pone.0328972)
Supplement: S2 Table — d – spacing of PbTe-HepA0.5/OA5.5 calculated from HRTEM images and its corresponding hkl index. (PDF) [file pone.0328972.s012.pdf]

S2 Table. d – spacing calculations. d – spacing of PbTe-HepA$_{0.5}$/OA$_{5.5}$ calculated from HRTEM images and its corresponding hkl index.

Original image	Zoom In	FFT function	Line plot function	Index hkl
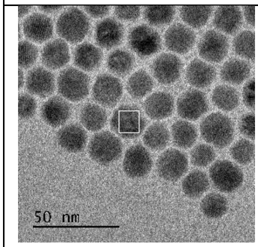	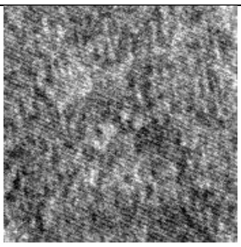	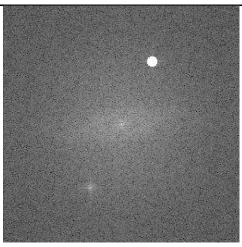	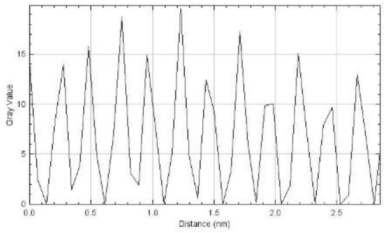	220<br><br>d = 0.233 nm
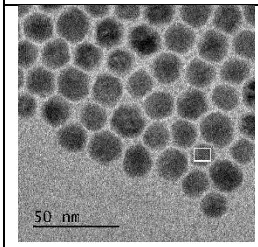	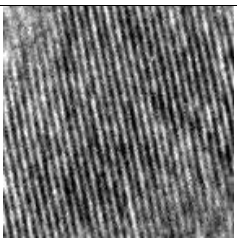	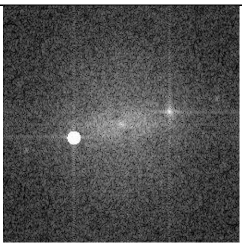	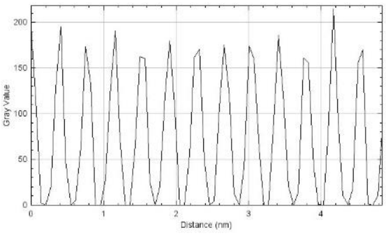	111<br><br>d = 0.372 nm
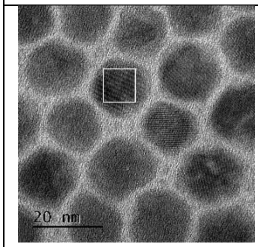	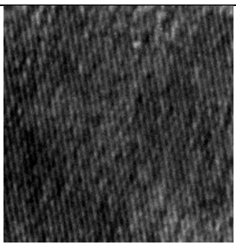	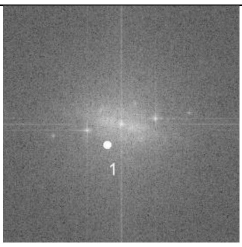	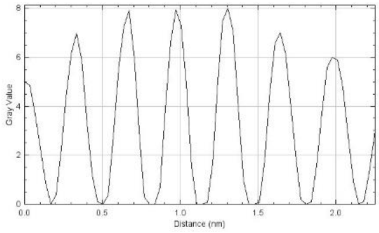	200<br><br>d = 0.321 nm
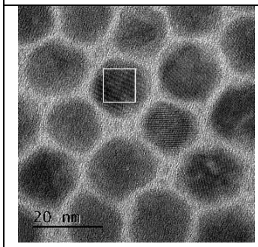	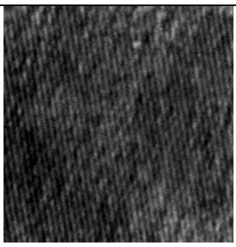	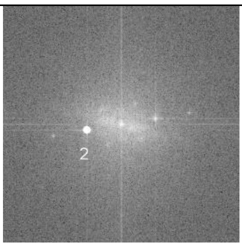	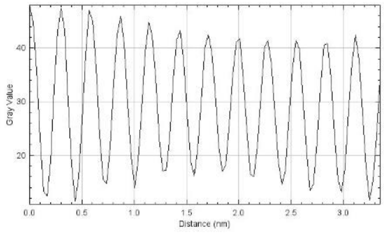	420<br><br>d = 0.280 /2 = 0.140 nm
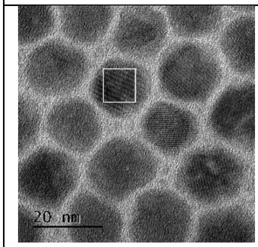	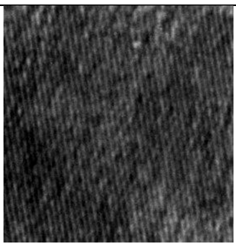	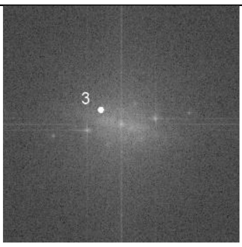	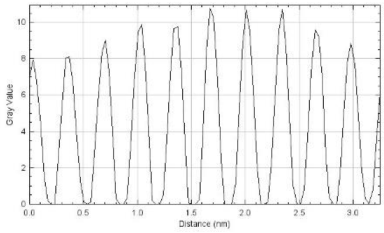	200<br><br>d = 0.328 nm
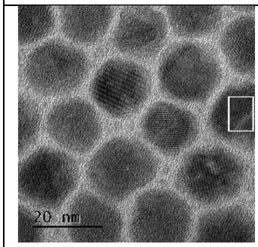	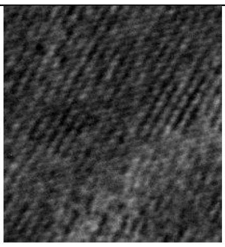	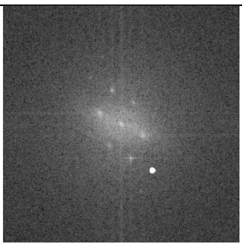	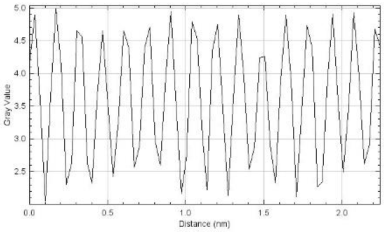	331<br><br>d = 0.149 nm

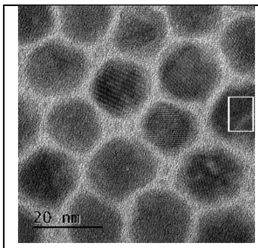	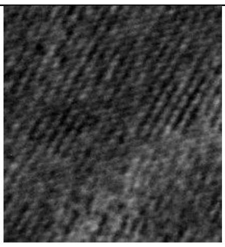	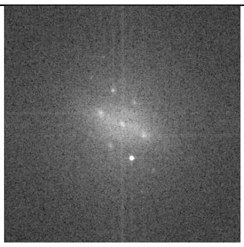	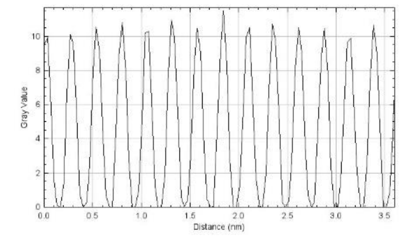	422<br>d = 0.257 /2= 0.129 nm
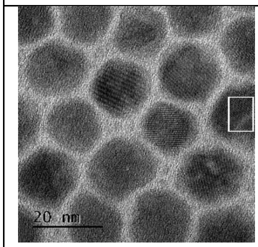	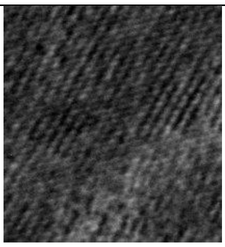	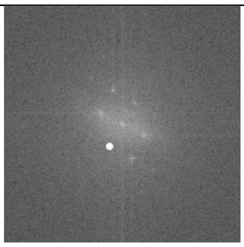	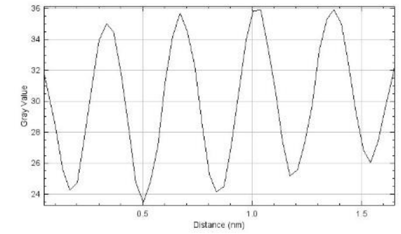	200<br><br>d = 0.320 nm
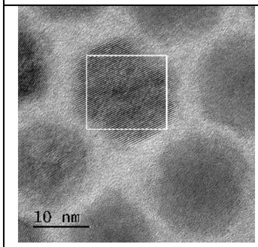	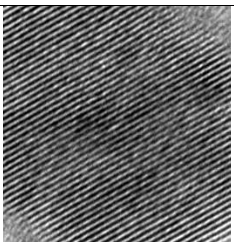	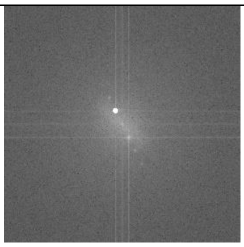	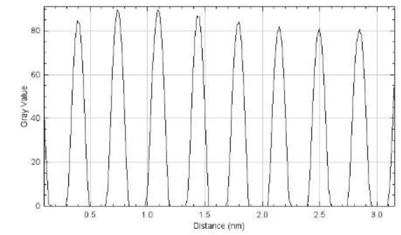	111<br><br>d = 0.377 nm
