# Supplementary material for: Carboxylic ligands and their influence on the structural properties of PbTe quantum dots
Source: PLoS One. 2025 Jul 31;20(7):e0328972. doi: 10.1371/journal.pone.0328972 (PMC12312907; doi:10.1371/journal.pone.0328972)
Supplement: S3 Table — d – spacing of PbTe-HepA1/OA5 calculated from HRTEM images and its corresponding hkl index. (PDF) [file pone.0328972.s013.pdf]

S3 Table : d – spacing calculations. d – spacing of PbTe-HepA$_1$/OA$_5$ calculated from HRTEM images and its corresponding hkl index.

Original image	Zoom In	FFT function	Line plot function	Index hkl
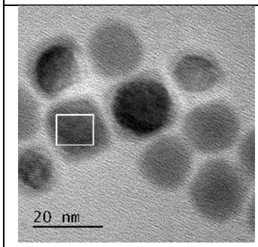	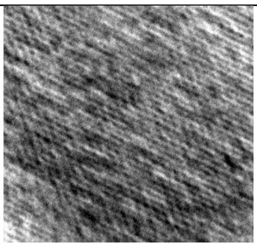	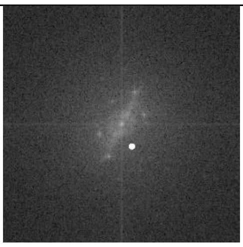	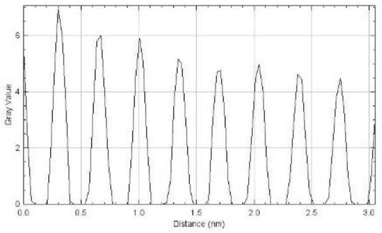	400<br>d = 0.332/2= 0.166 nm
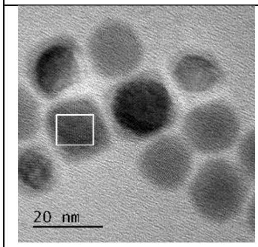	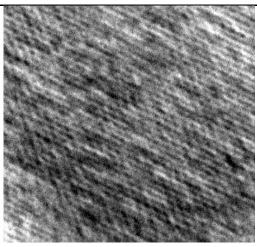	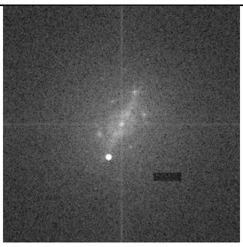	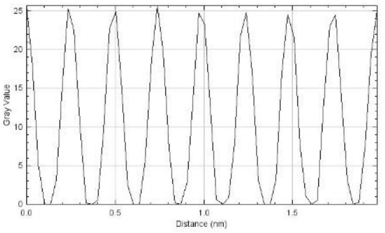	511<br>d = 0.247/2= 0.124 nm
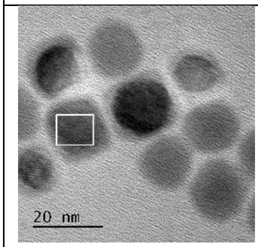	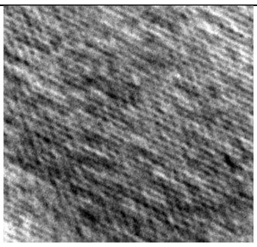	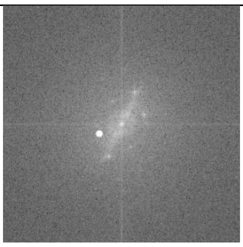	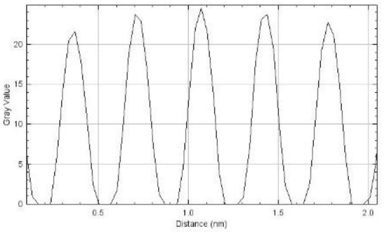	200<br>d = 0.325 nm
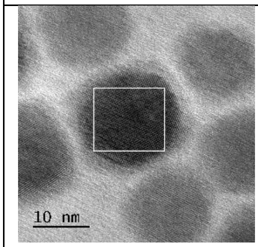	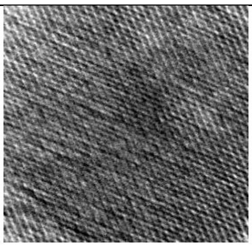	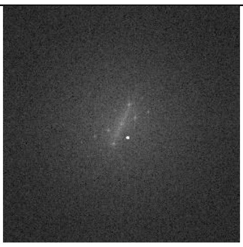	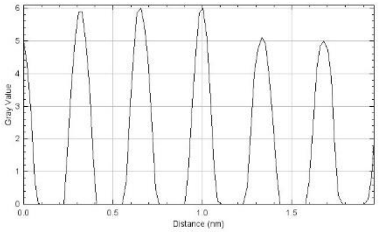	200<br>d = 0.326 nm
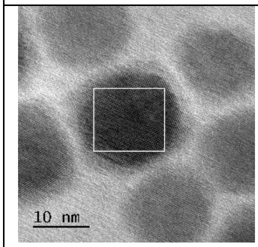	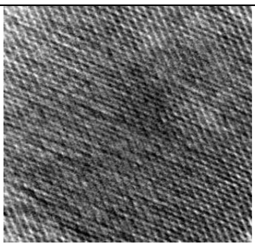	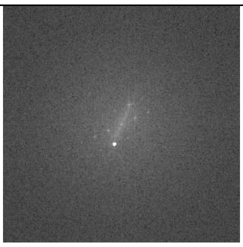	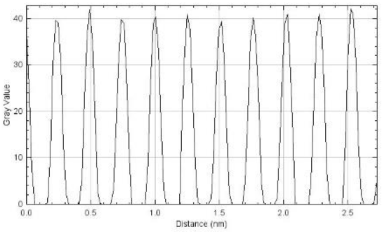	511<br>d = 0.248/2= 0.124 nm
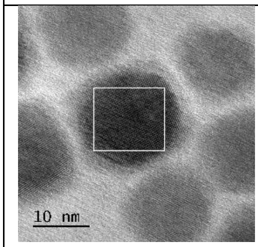	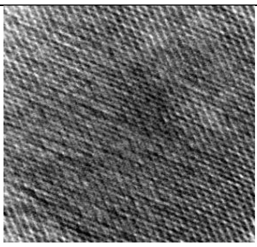	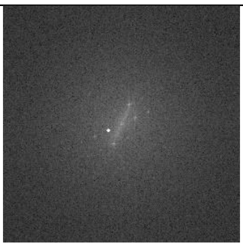	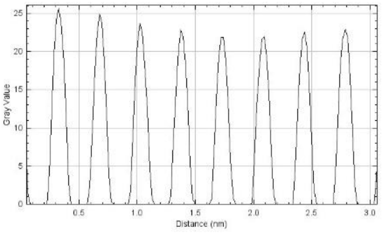	400<br>d = 0.334/2= 0.167 nm

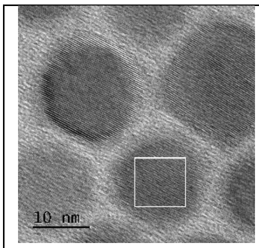	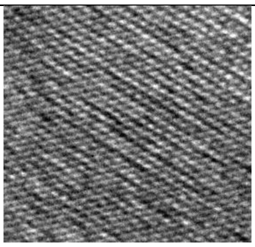	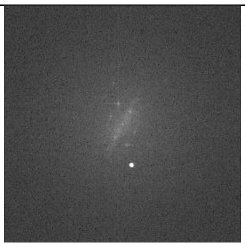	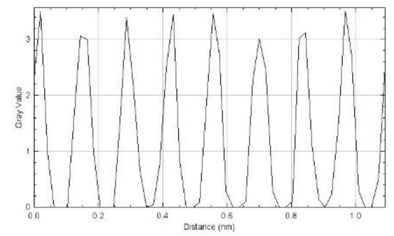	422<br><br>d = 0.136 nm
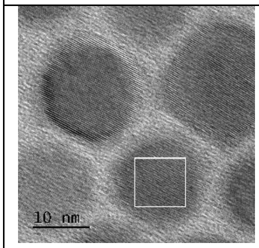	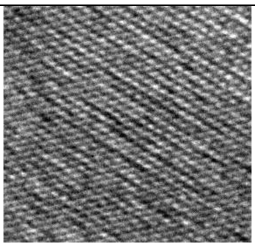	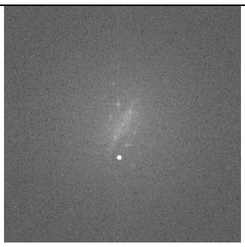	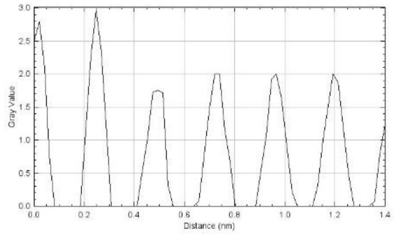	220<br><br>d = 0.233 nm
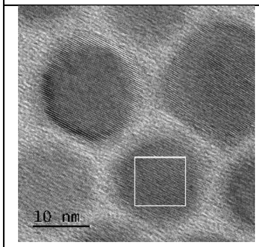	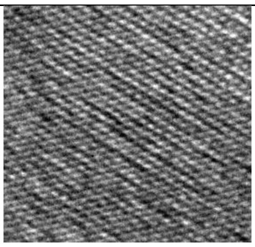	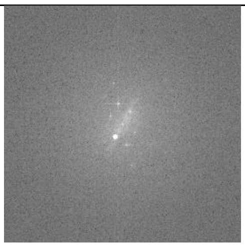	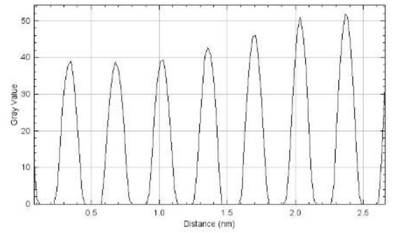	200<br><br>d = 0.322 nm
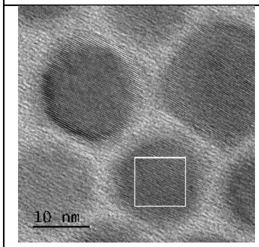	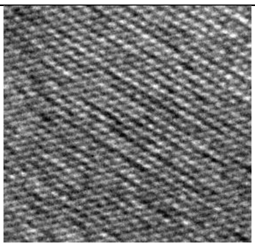	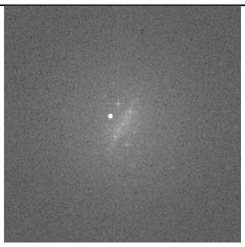	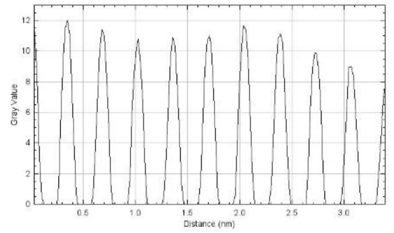	111<br><br>d = 0.373 mn
