# Supplementary material for: Carboxylic ligands and their influence on the structural properties of PbTe quantum dots
Source: PLoS One. 2025 Jul 31;20(7):e0328972. doi: 10.1371/journal.pone.0328972 (PMC12312907; doi:10.1371/journal.pone.0328972)
Supplement: S4 Table — d – spacing of PbTe-HepA1.5/OA4.5 calculated from HRTEM images and its corresponding hkl index. (PDF) [file pone.0328972.s014.pdf]

S4 Table. d – spacing calculations. d – spacing of PbTe-HepA$_{1.5}$/OA$_{4.5}$ calculated from HRTEM images and its corresponding hkl index.

Original image	Zoom In	FFT function	Line plot function	Index hkl
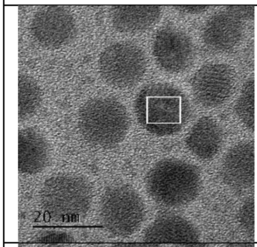	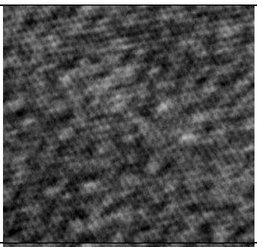	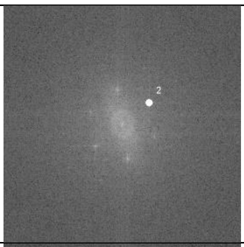	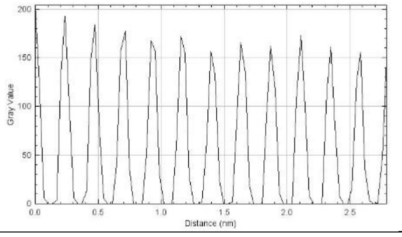	220<br>d = 0.230 nm
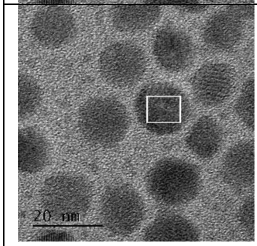	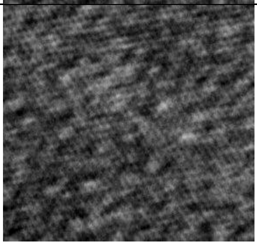	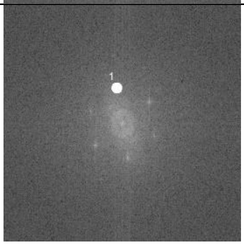	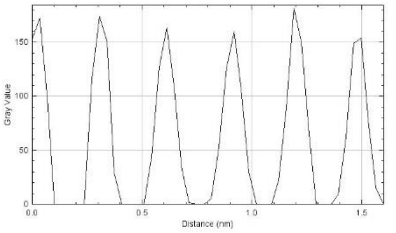	200<br>d = 0.320 nm
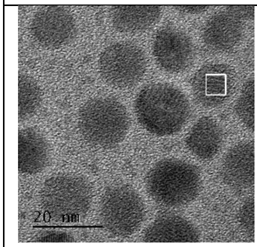	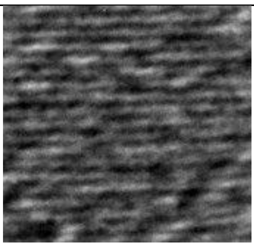	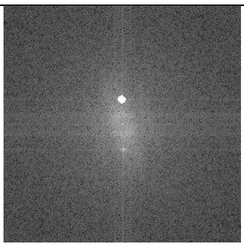	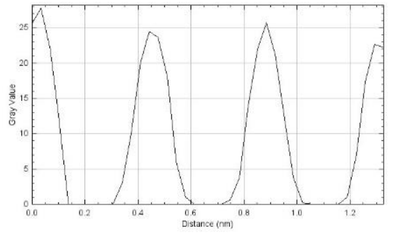	111<br>d = 0.370 nm
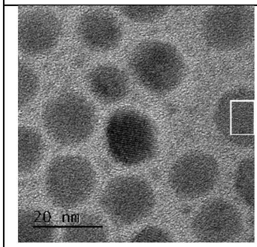	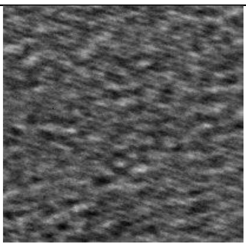	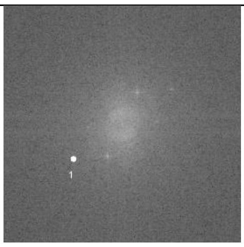	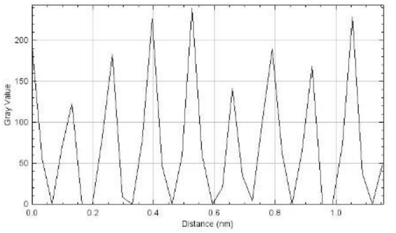	422<br>d = 0.129 nm
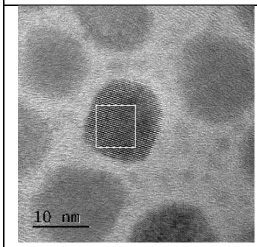	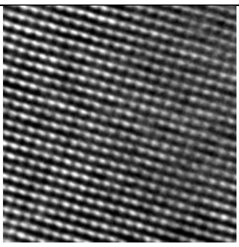	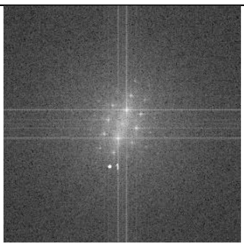	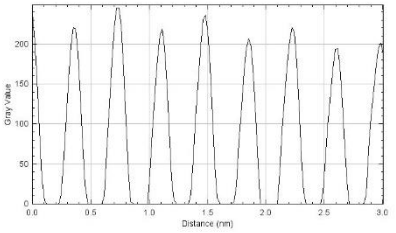	111<br>d = 0.375 nm
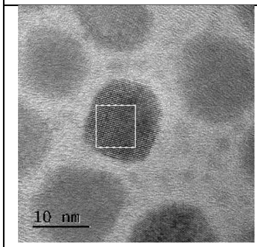	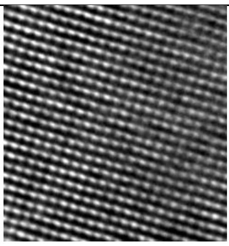	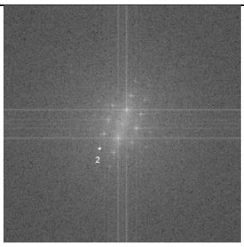	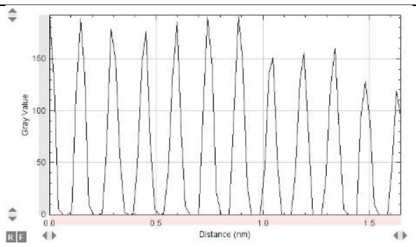	331<br>d = 0.150 nm

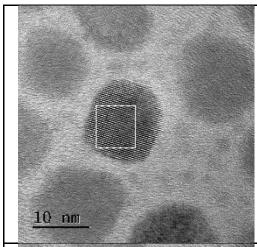	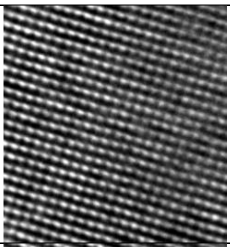	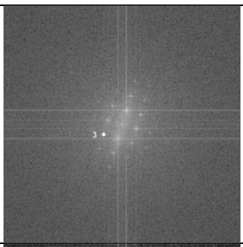	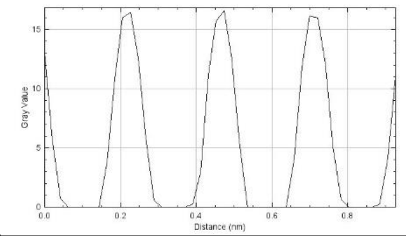	200<br><br>d = 0.321 nm
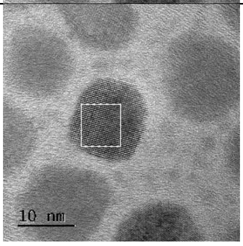	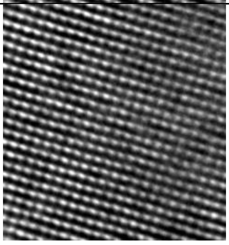	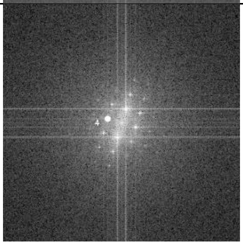	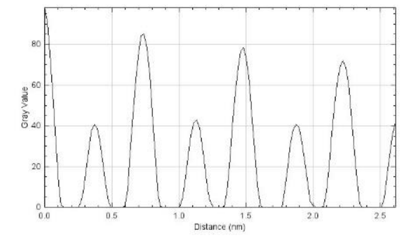	111<br><br>d = 0.372 nm
