# Supplementary material for: Carboxylic ligands and their influence on the structural properties of PbTe quantum dots
Source: PLoS One. 2025 Jul 31;20(7):e0328972. doi: 10.1371/journal.pone.0328972 (PMC12312907; doi:10.1371/journal.pone.0328972)
Supplement: S5 Table — d – spacing of PbTe-HepA2/OA4 calculated from HRTEM images and its corresponding hkl index. (PDF) [file pone.0328972.s015.pdf]

S5 Table. d – spacing calculations. d – spacing of PbTe-HepA_2/OA_4 calculated from HRTEM images and its corresponding hkl index.

Original image	Zoom In	FFT function	Line plot function	Index hkl
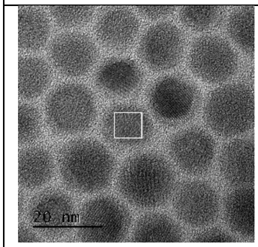	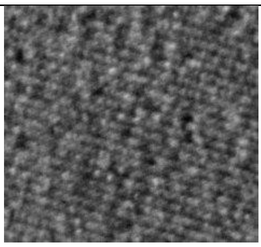	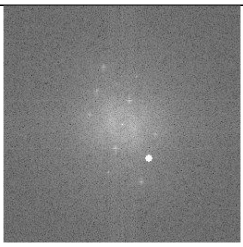	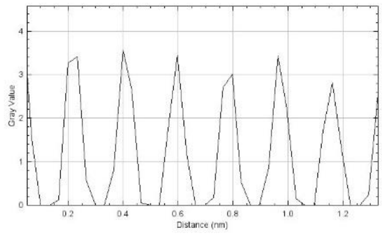	222<br>d = 0.182 nm
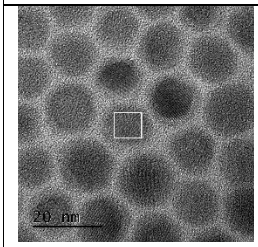	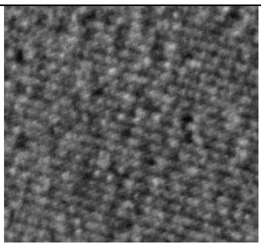	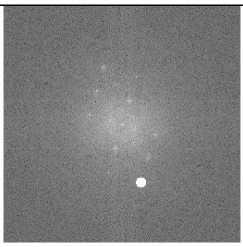	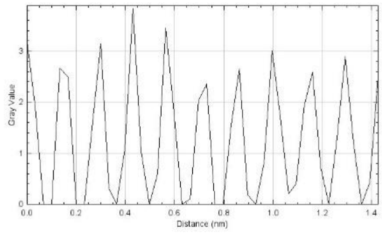	420<br>d = 0.142 nm
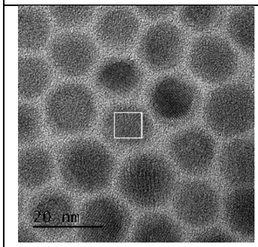	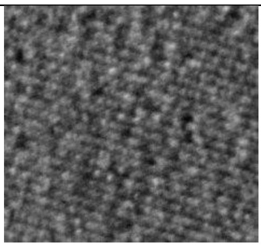	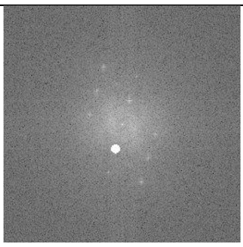	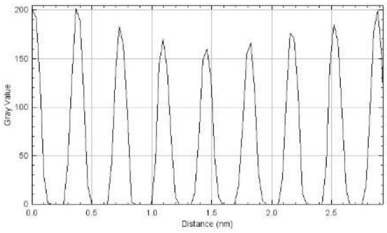	111<br>d = 0.366 nm
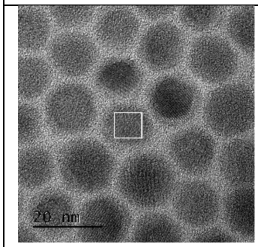	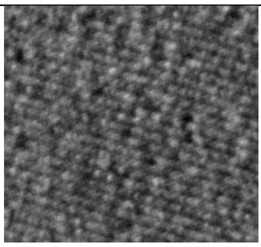	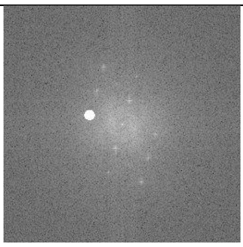	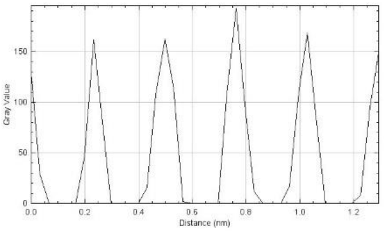	422<br>d = 0.258 /2 = 0.129 nm
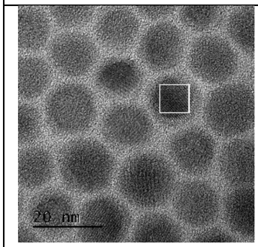	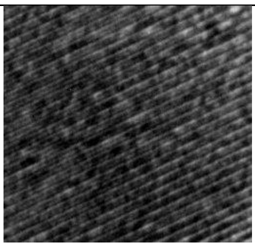	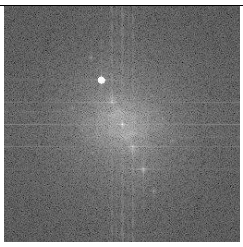	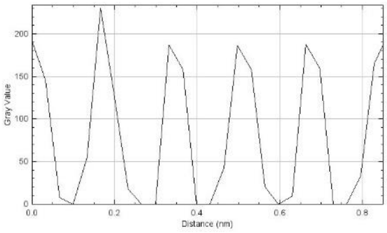	400<br>d = 0.160 nm
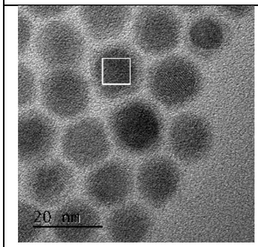	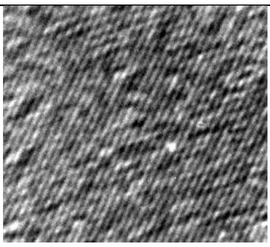	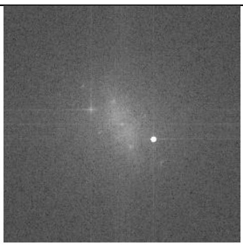	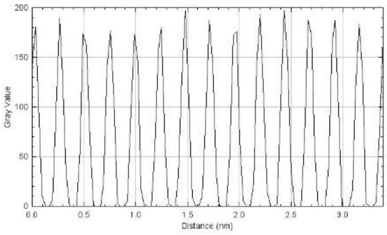	511<br>d = 0.242/ 2 = 0.121 nm

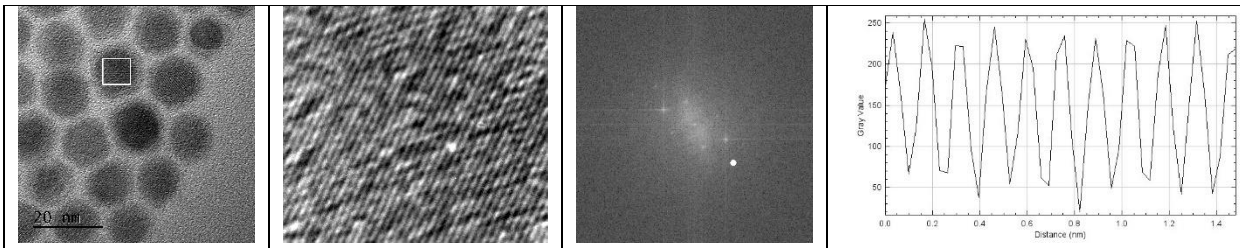	331<br><br>d = 0.148 nm
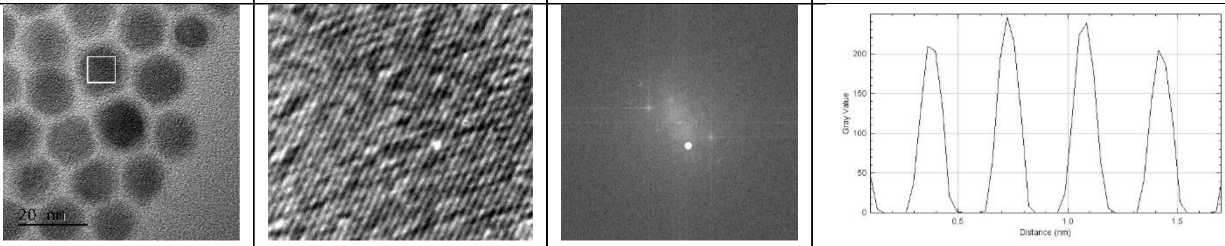	200<br><br>d = 0.320 nm
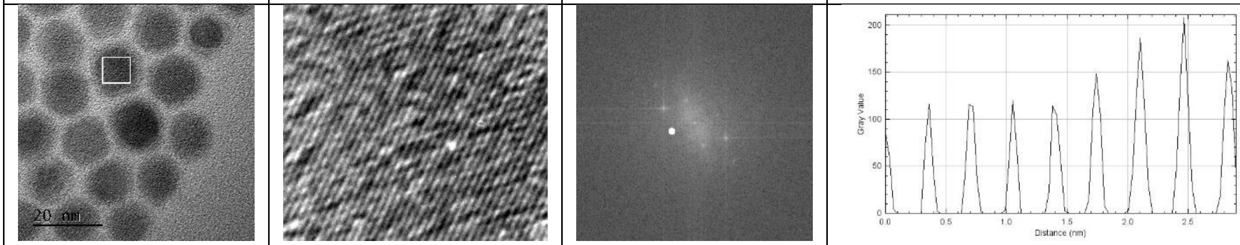	222<br>d = 0.361/2 = 0.181 nm
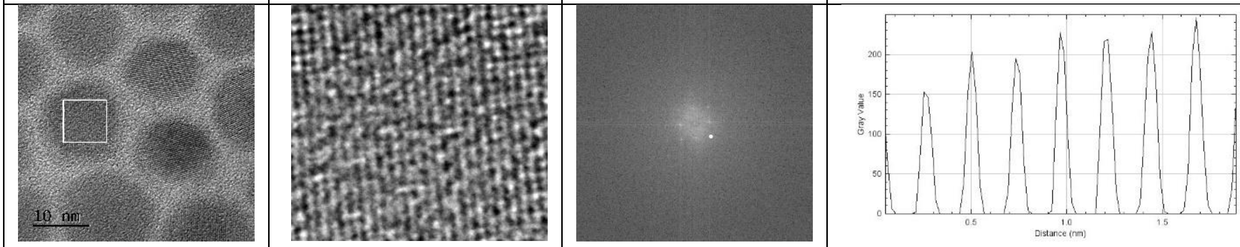	220<br><br>d = 0.229 nm
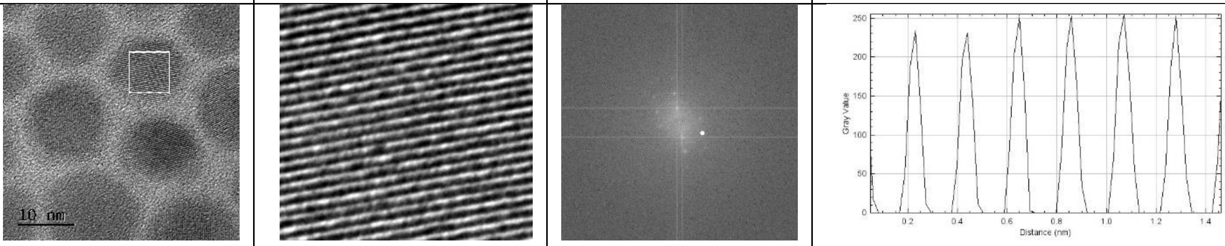	311<br>d = 0.196 nm
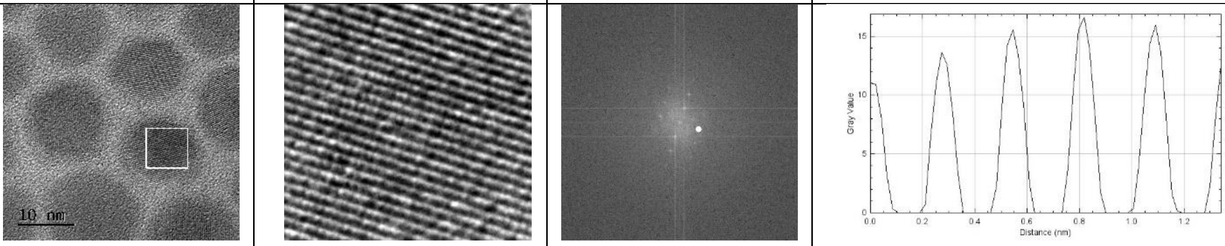	422<br><br>d = 0.268 /2 = 0.134 nm
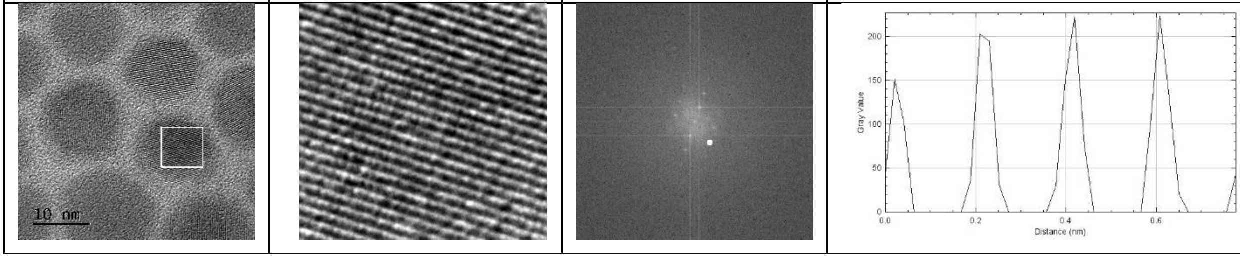	311<br><br>d = 0.194 nm

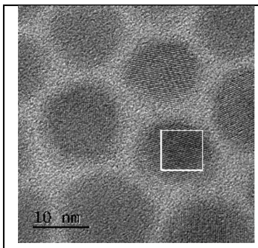	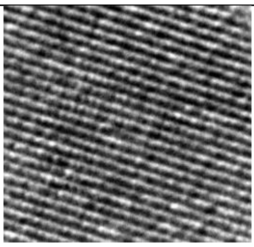	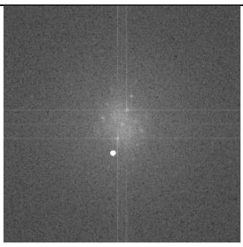	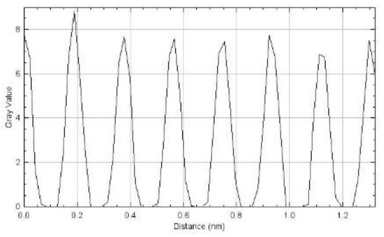	222<br><br>d = 0.188 nm
