# Supplementary material for: Carboxylic ligands and their influence on the structural properties of PbTe quantum dots
Source: PLoS One. 2025 Jul 31;20(7):e0328972. doi: 10.1371/journal.pone.0328972 (PMC12312907; doi:10.1371/journal.pone.0328972)
Supplement: S6 Table — d – spacing of PbTe-HexA0.5/OA5.5 calculated from HRTEM images and its corresponding hkl index. (PDF) [file pone.0328972.s016.pdf]

S6 Table. d – spacing calculations. d – spacing of PbTe-HexA$_{0.5}$/OA$_{5.5}$ calculated from HRTEM images and its corresponding hkl index.

Original image	Zoom In	FFT function	Line plot function	Index hkl
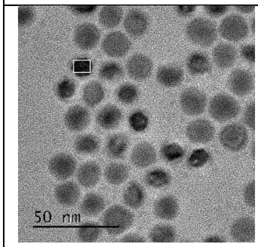	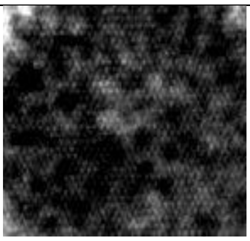	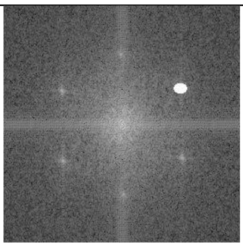	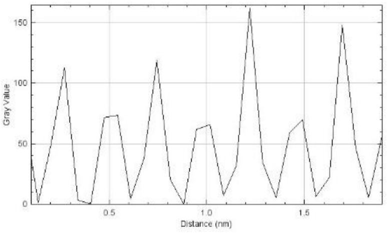	220<br>d = 0.224 nm
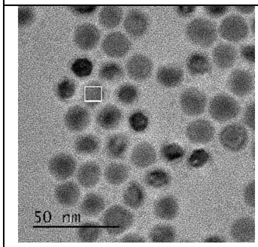	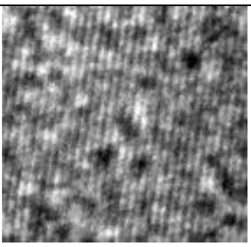	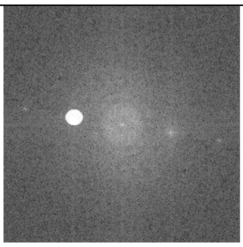	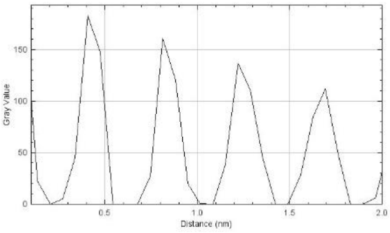	111<br>d = 0.380 nm
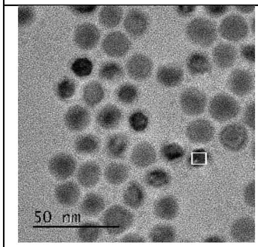	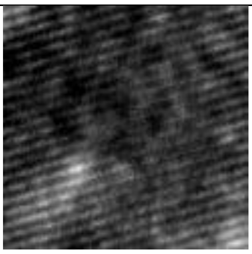	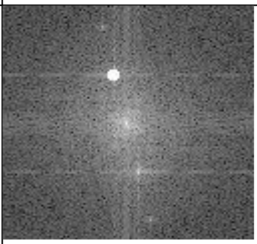	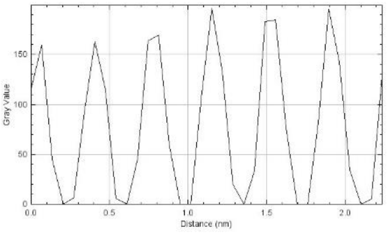	111<br>d = 0.373 nm
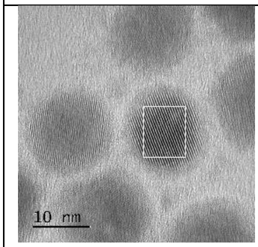	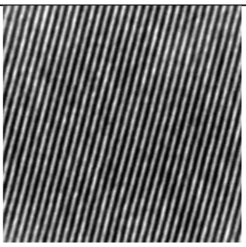	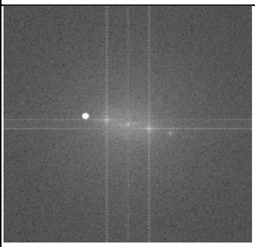	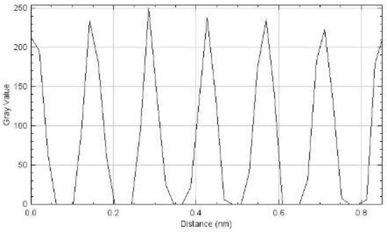	420<br>d = 0.142 nm
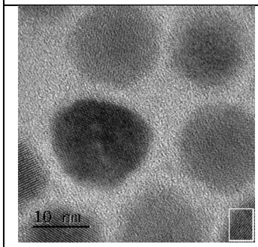	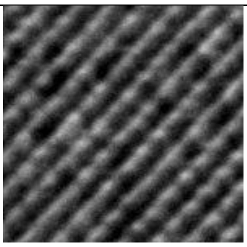	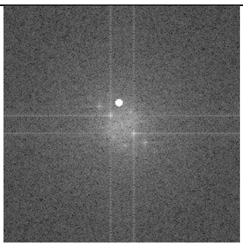	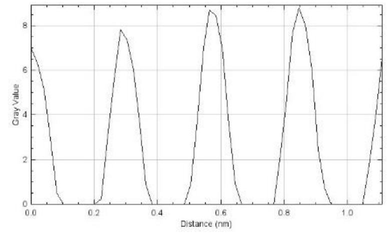	220<br>d = 0.222 nm
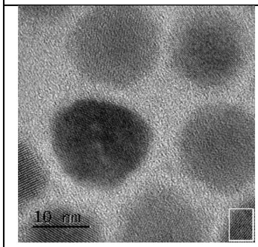	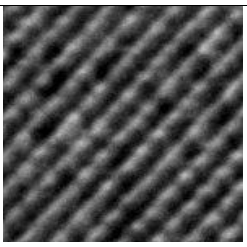	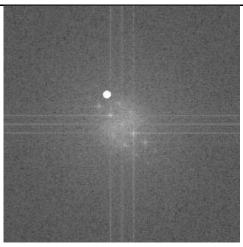	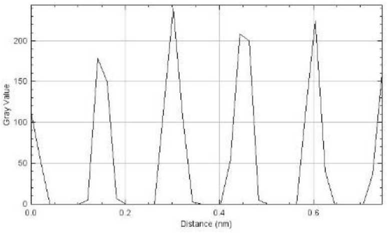	331<br>d = 0.149 nm

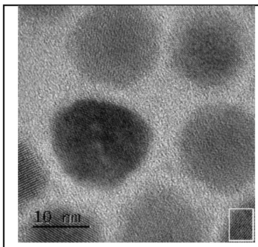	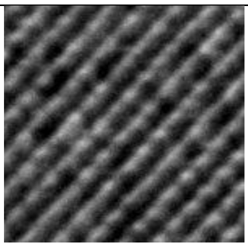	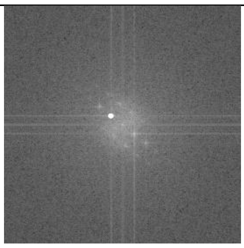	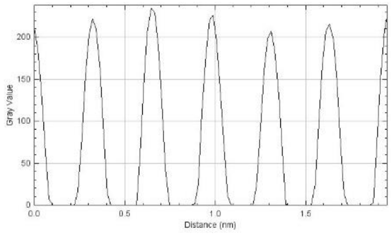	200<br><br>d = 0.325 nm
