# Supplementary material for: Carboxylic ligands and their influence on the structural properties of PbTe quantum dots
Source: PLoS One. 2025 Jul 31;20(7):e0328972. doi: 10.1371/journal.pone.0328972 (PMC12312907; doi:10.1371/journal.pone.0328972)
Supplement: S7 Table — d – spacing of PbTe-HexA1/OA5 calculated from HRTEM images and its corresponding hkl index. (PDF) [file pone.0328972.s017.pdf]

S7 Table. d – spacing calculations. d – spacing of PbTe-HexA$_1$/OA$_5$ calculated from HRTEM images and its corresponding hkl index.

Original image	Zoom In	FFT function	Line plot function	Index hkl
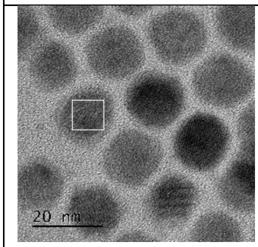	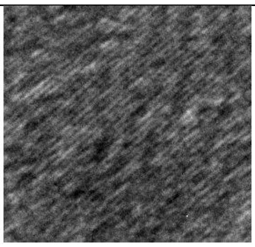	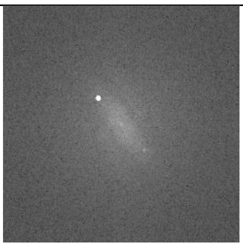	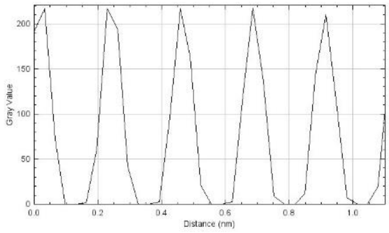	220<br>d = 0.223 nm
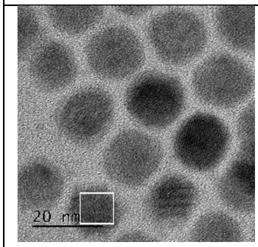	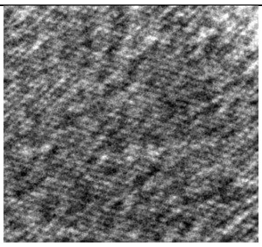	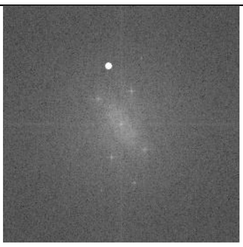	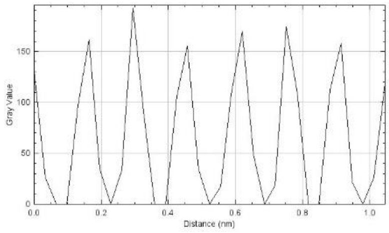	331<br>d = 0.149 nm
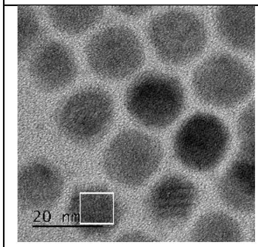	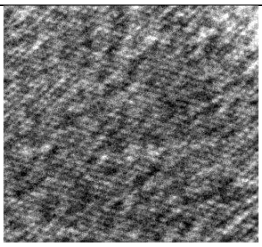	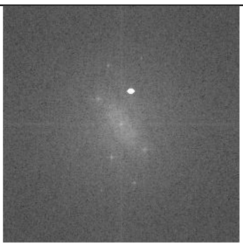	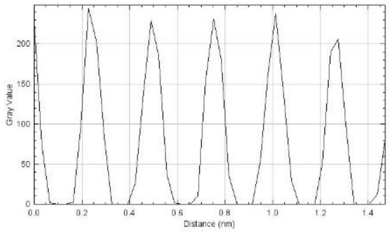	511<br>d = 0.245/2 = 0.123 nm
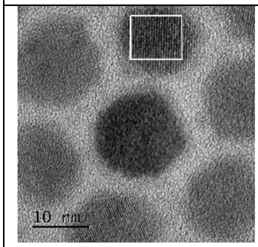	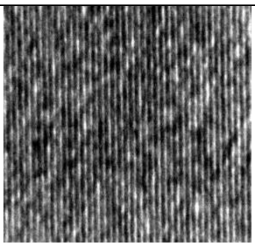	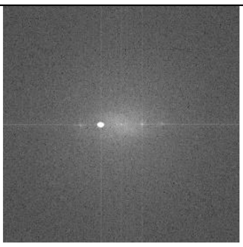	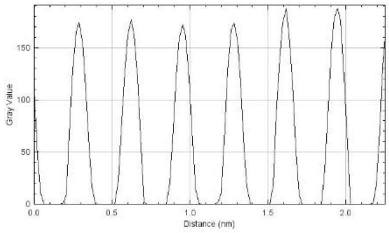	200<br>d = 0.321 nm
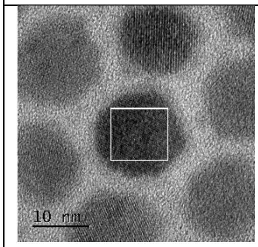	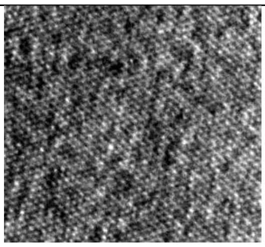	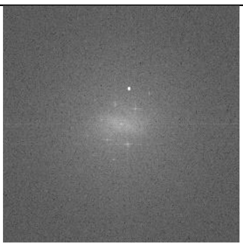	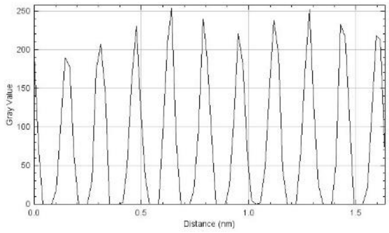	400<br>d = 0.163 nm
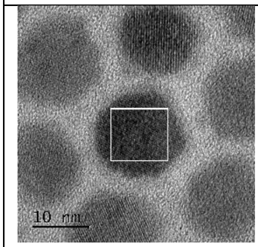	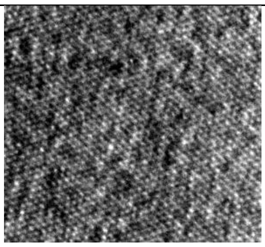	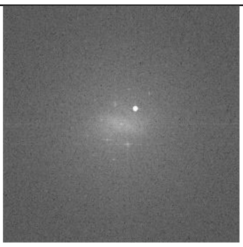	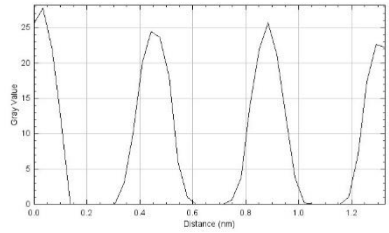	220<br>d = 0.233 nm

<table>
<tr>
<td>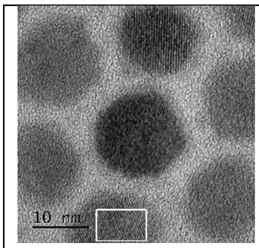</td>
<td>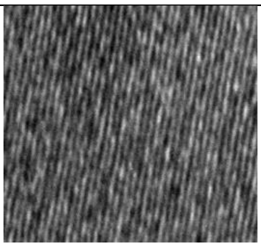</td>
<td>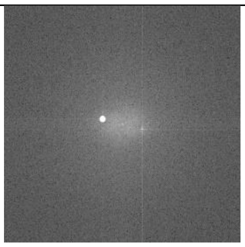</td>
<td>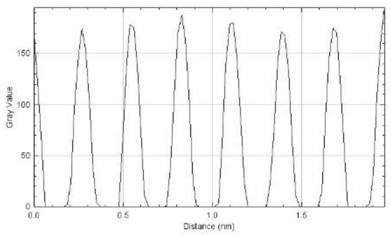</td>
<td>420

d = 0.282/2 =0.141 nm</td>
</tr>
</table>
