# Supplementary material for: Carboxylic ligands and their influence on the structural properties of PbTe quantum dots
Source: PLoS One. 2025 Jul 31;20(7):e0328972. doi: 10.1371/journal.pone.0328972 (PMC12312907; doi:10.1371/journal.pone.0328972)
Supplement: S8 Table — d – spacing of PbTe-HexA1.5/OA4.5 calculated from HRTEM images and its corresponding hkl index. (PDF) [file pone.0328972.s018.pdf]

S8 Table. d – spacing calculations. d – spacing of PbTe-HexA$_{1.5}$/OA$_{4.5}$ calculated from HRTEM images and its corresponding hkl index.

Original image	Zoom In	FFT function	Line plot function	Index hkl
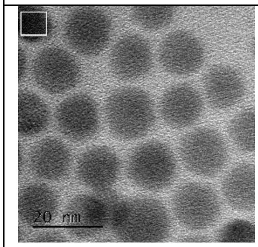	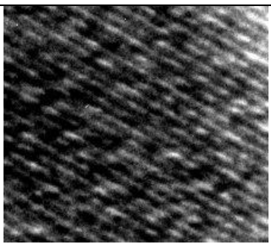	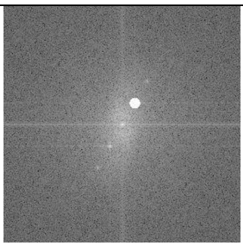	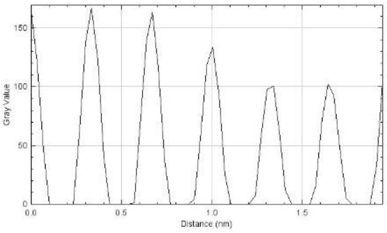	200 d = 0.323 nm
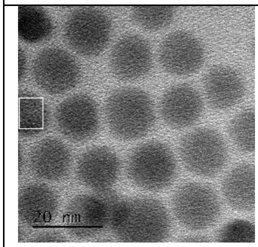	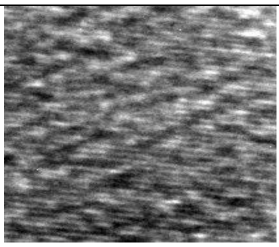	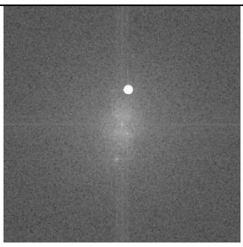	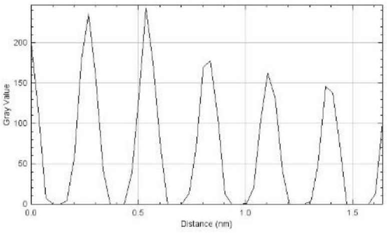	422 d = 0.273/2 = 0.136 nm
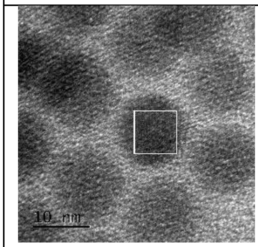	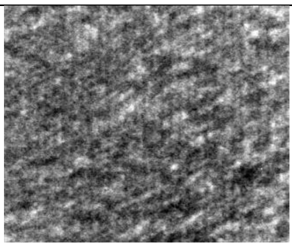	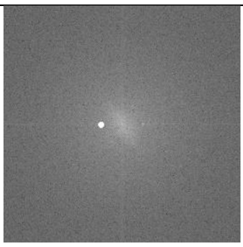	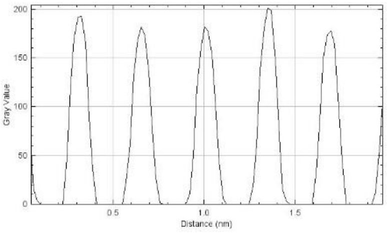	200 d = 0.321 nm
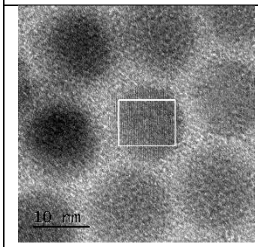	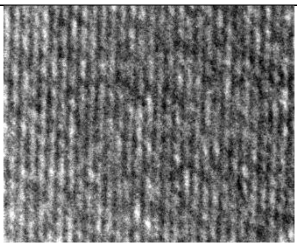	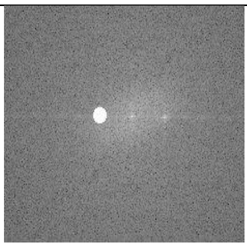	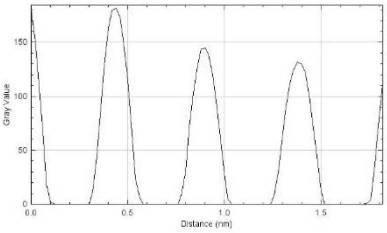	220 d = 0.227 nm
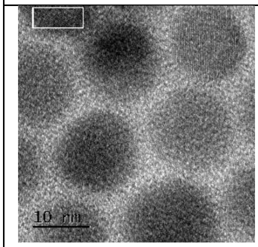	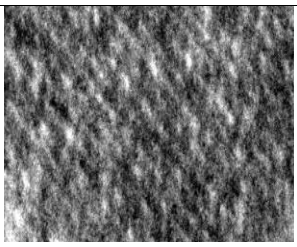	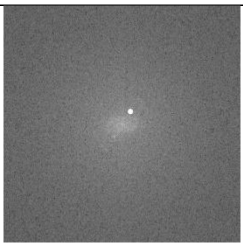	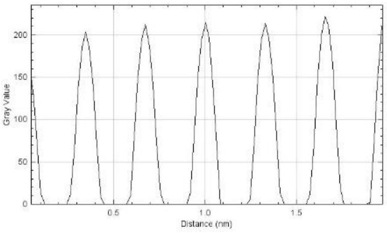	200 d = 0.320 nm
