# Supplementary material for: Carboxylic ligands and their influence on the structural properties of PbTe quantum dots
Source: PLoS One. 2025 Jul 31;20(7):e0328972. doi: 10.1371/journal.pone.0328972 (PMC12312907; doi:10.1371/journal.pone.0328972)
Supplement: S9 Table — d – spacing of PbTe-HexA2/OA4 calculated from HRTEM images and its corresponding hkl index. (PDF) [file pone.0328972.s019.pdf]

S9 Table. d – spacing calculations. d – spacing of PbTe-HexA$_2$/OA$_4$ calculated from HRTEM images and its corresponding hkl index.

Original image	Zoom In	FFT function	Line plot function	Index hkl
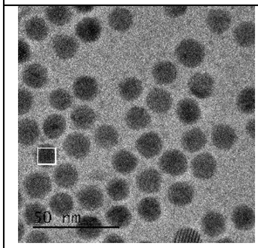	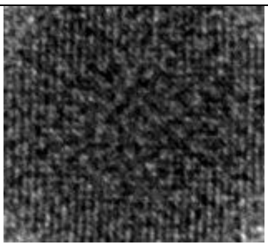	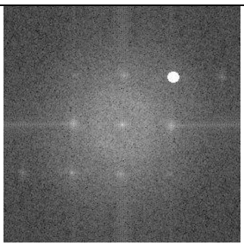	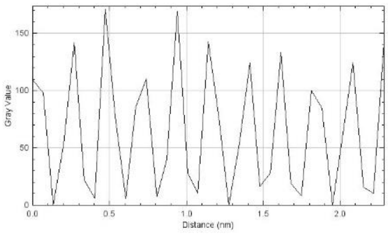	220 d = 0.228 nm
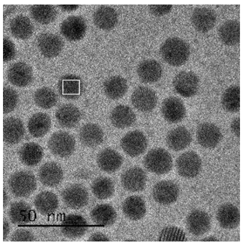	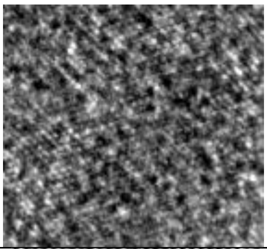	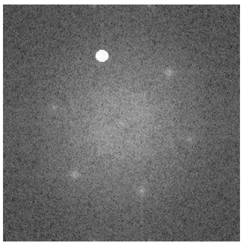	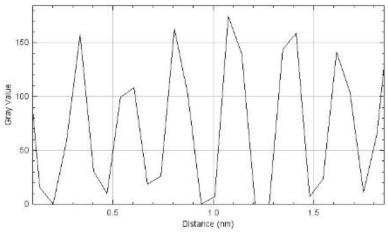	511 d = 0.250/2 = 0.125 nm
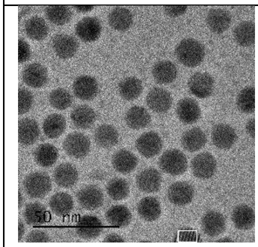	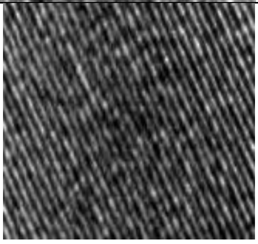	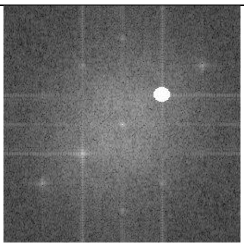	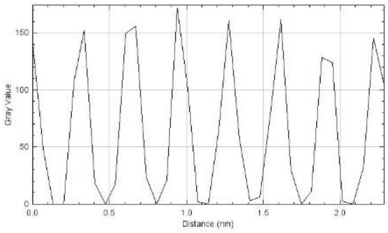	200 d = 0.325 nm
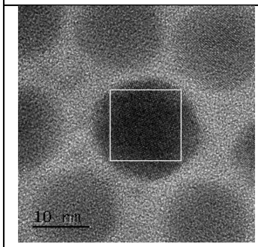	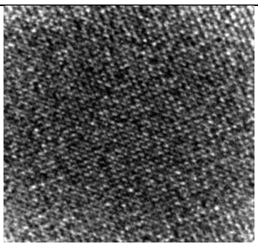	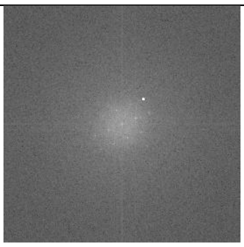	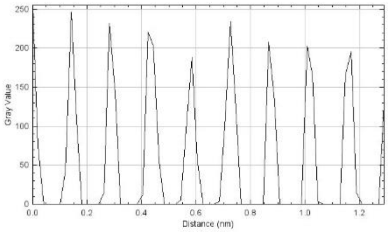	420 d = 0.143 nm
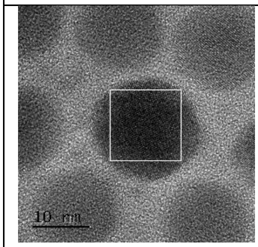	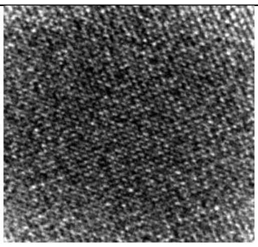	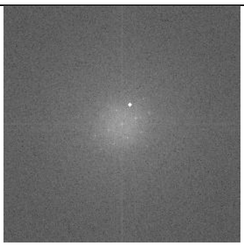	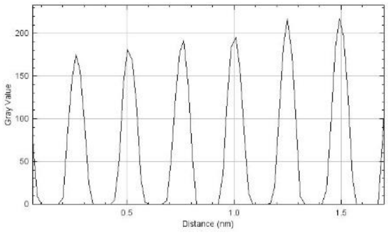	220 d = 0.234 nm
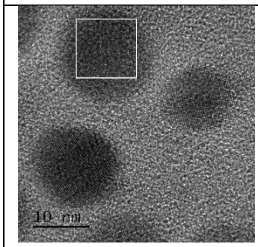	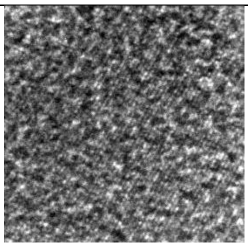	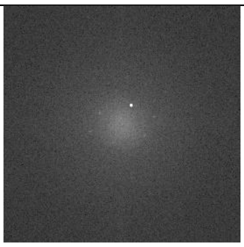	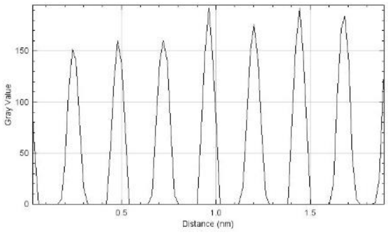	220 d = 0.232 nm
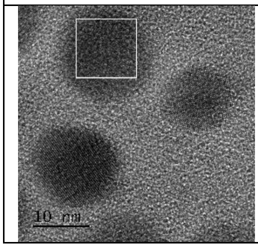	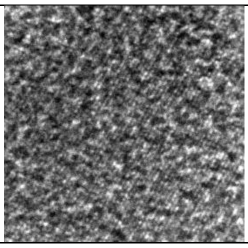	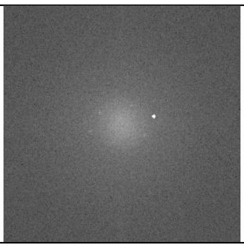	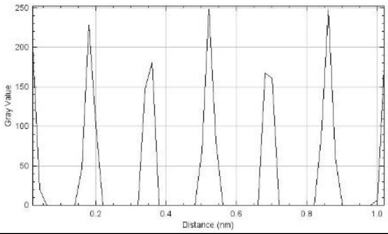	400 d = 0.166 nm

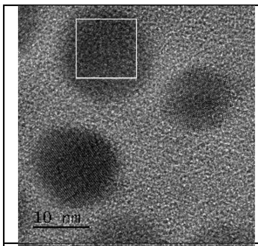	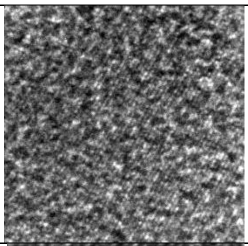	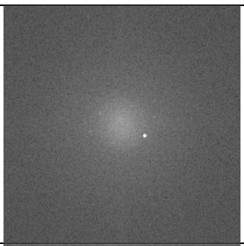	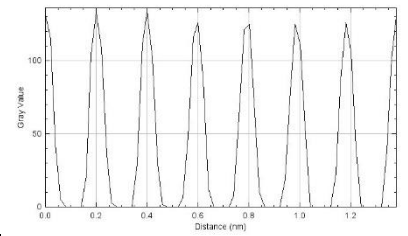	311<br><br>d = 0.197 nm
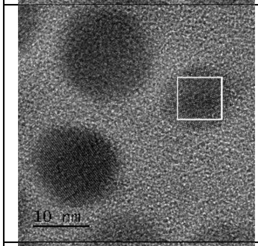	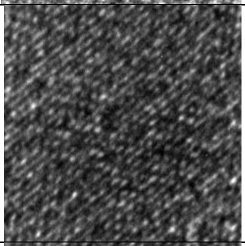	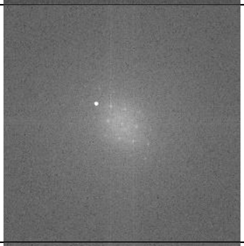	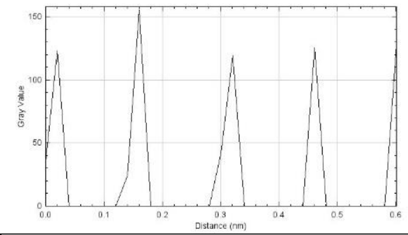	331<br><br>d = 0.150 nm
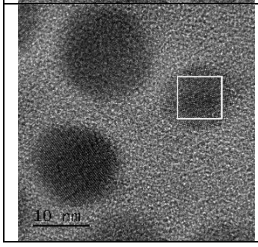	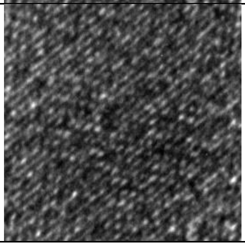	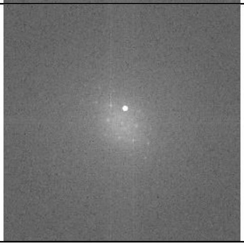	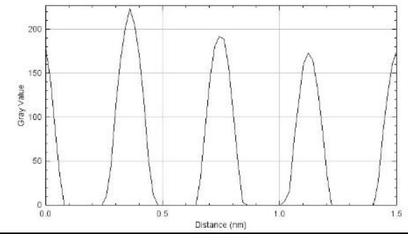	111<br><br>d = 0.375 nm
