# Supplementary material for: Carboxylic ligands and their influence on the structural properties of PbTe quantum dots
Source: PLoS One. 2025 Jul 31;20(7):e0328972. doi: 10.1371/journal.pone.0328972 (PMC12312907; doi:10.1371/journal.pone.0328972)
Supplement: S10 Table — d – spacing of PbTe-AcA1/OA5 calculated from HRTEM images and its corresponding hkl index. (PDF) [file pone.0328972.s020.pdf]

S10 Table. d – spacing calculations. d – spacing of PbTe-AcA$_1$/OA$_5$ calculated from HRTEM images and its corresponding hkl index.

Original image	Zoom In	FFT function	Line plot function	Index hkl
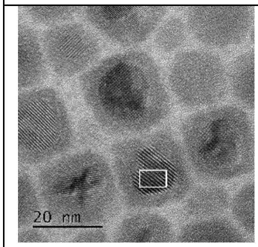	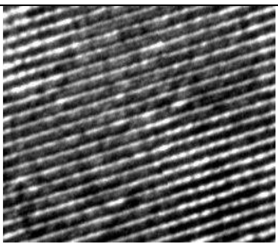	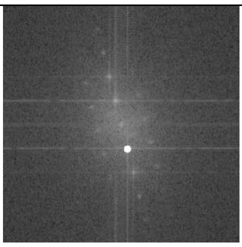	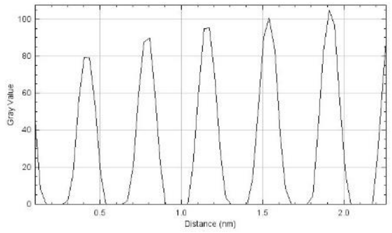	111 d = 0.376 nm
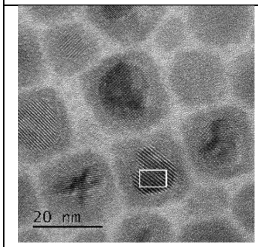	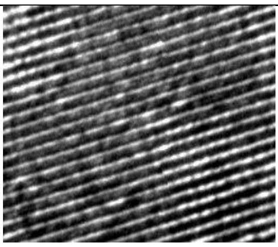	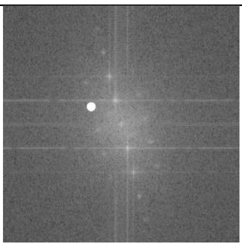	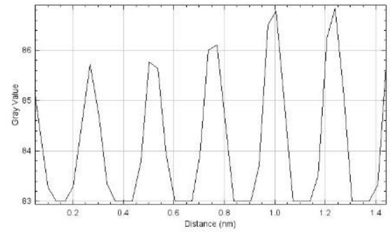	220 d = 0.231 nm
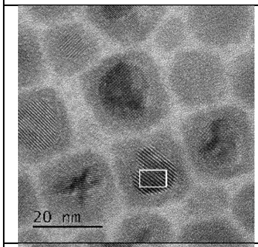	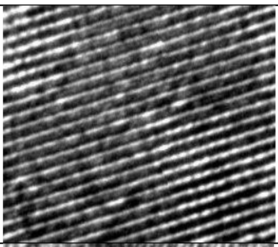	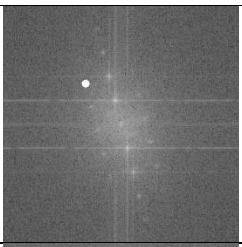	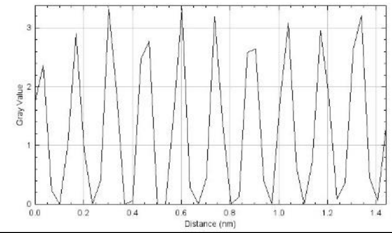	420 d = 0.144 nm
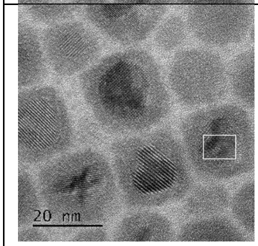	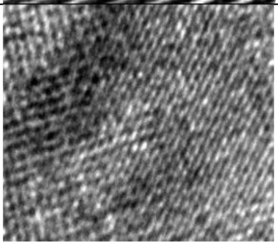	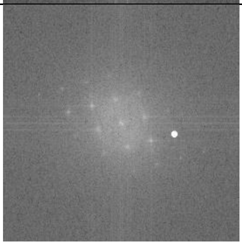	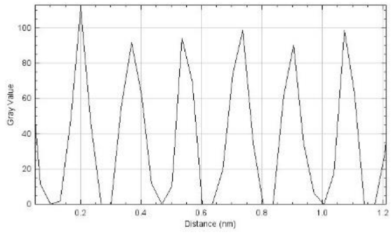	400 d = 0.165 nm
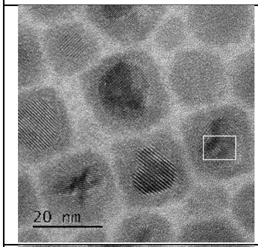	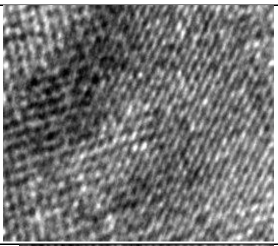	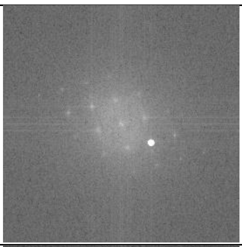	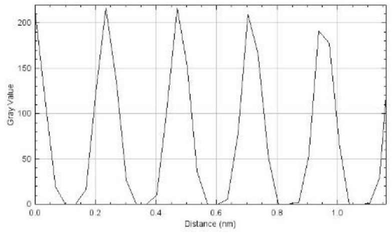	200 d = 0.320 nm
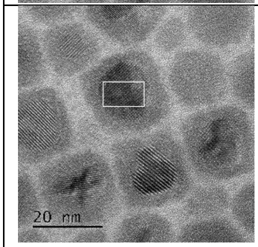	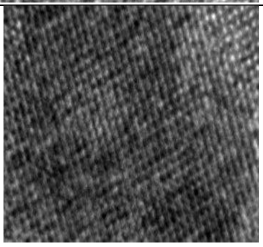	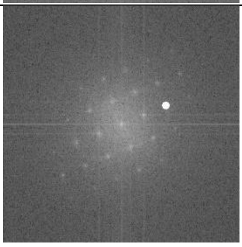	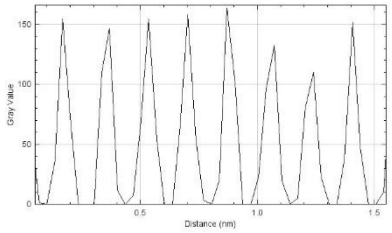	400 d = 0.166 nm
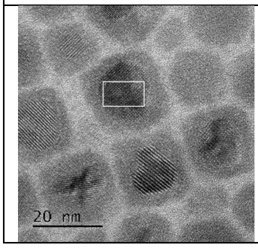	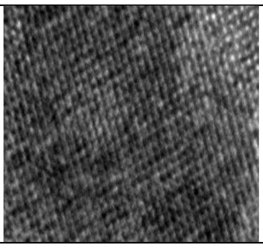	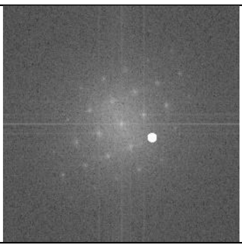	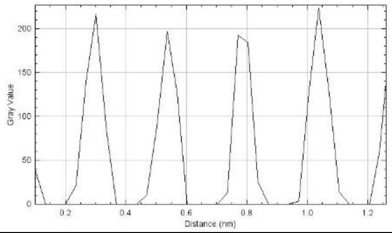	220 d = 0.232 nm

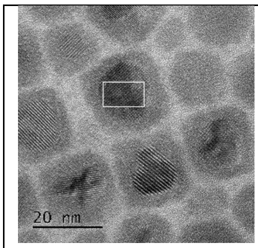	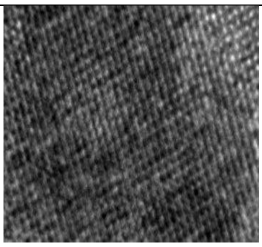	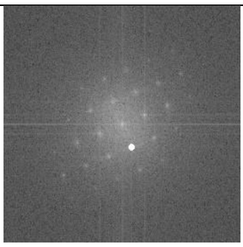	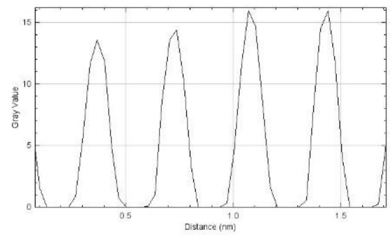	200<br>d =<br>0.326<br>nm
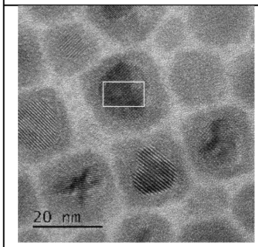	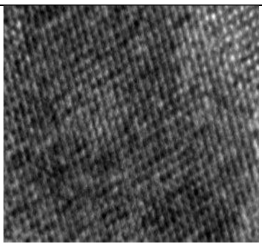	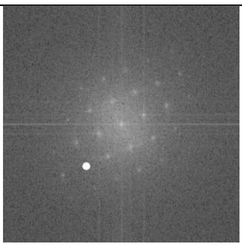	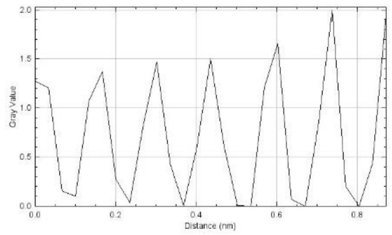	420<br>d =<br>0.145<br>nm
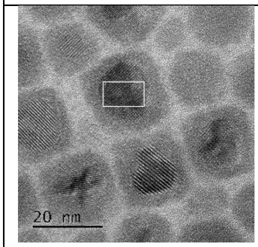	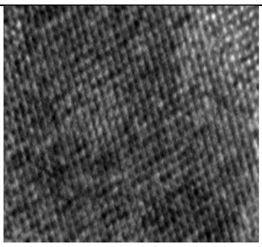	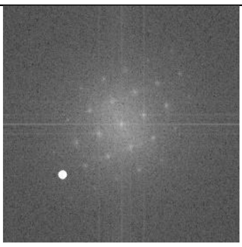	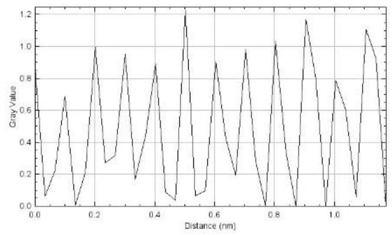	531<br>d =<br>0.106<br>nm
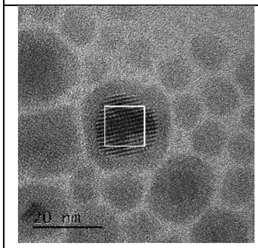	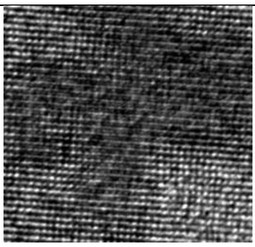	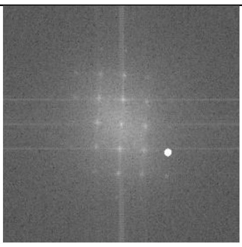	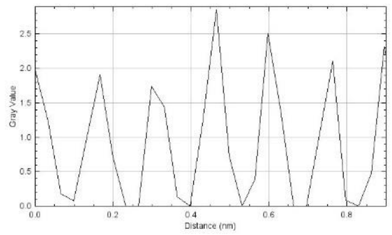	331<br>d =<br>0.150<br>nm
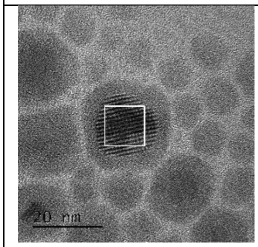	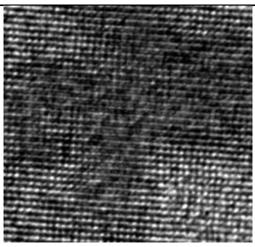	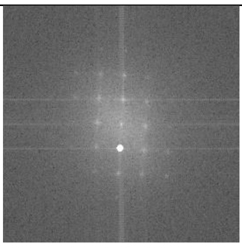	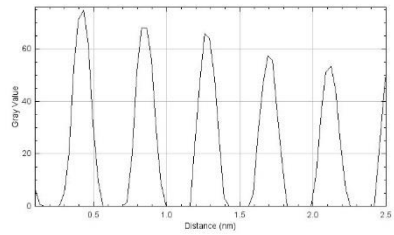	311<br>d =<br>0.200<br>nm
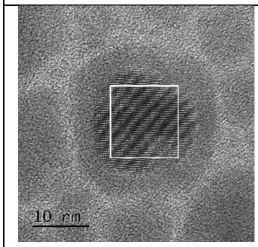	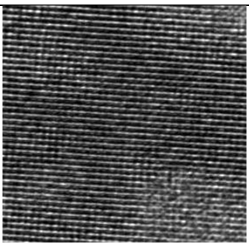	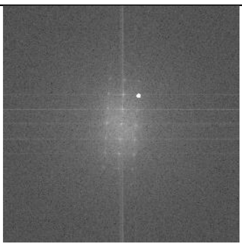	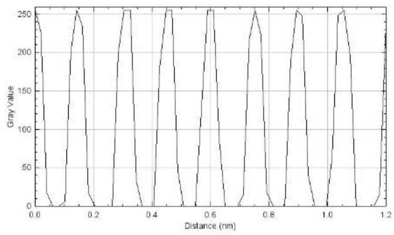	331<br>d =<br>0.150<br>nm

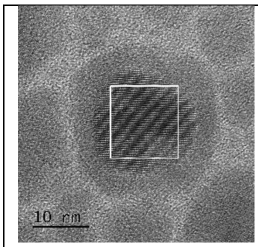	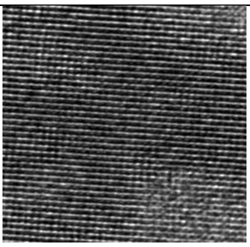	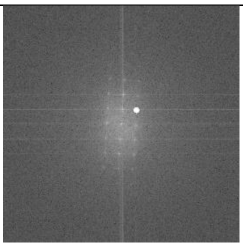	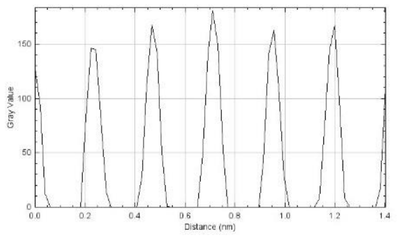	220<br>d =<br>0.233<br>nm
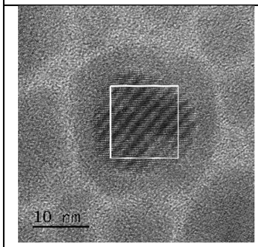	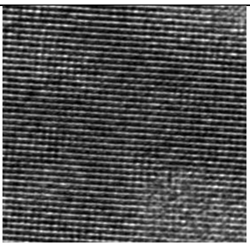	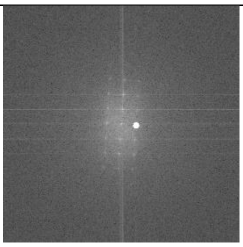	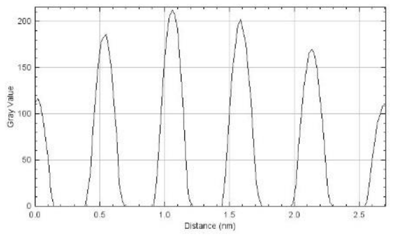	422<br>d =<br>0.540<br>/2=<br>0.270<br>/2=<br>0.135<br>nm
